# Steroid Alkaloids from *Holarrhena africana* with Strong Activity against *Trypanosoma brucei rhodesiense*

**DOI:** 10.3390/molecules22071129

**Published:** 2017-07-06

**Authors:** Charles Okeke Nnadi, Ngozi Justina Nwodo, Marcel Kaiser, Reto Brun, Thomas J. Schmidt

**Affiliations:** 1 Institute of Pharmaceutical Biology and Phytochemistry (IPBP), University of Münster, PharmaCampus Corrensstraße 48, D-48149 Münster, Germany; charles.nnadi@unn.edu.ng; 2 Department of Pharmaceutical and Medicinal Chemistry, Faculty of Pharmaceutical Sciences, University of Nigeria Nsukka, Enugu State 410001, Nigeria; ngozi.nwodo@unn.edu.ng; 3 Swiss Tropical and Public Health Institute (Swiss TPH), Socinstr. 57, CH-4051 Basel, Switzerland; marcel.kaiser@unibas.ch (M.K.); reto.brun@unibas.ch (R.B.); 4 University of Basel, Petersplatz 1, CH-4003 Basel, Switzerland

**Keywords:** *Holarrhena africana*, *Holarrhena floribunda*, steroid alkaloid, *Trypanosoma brucei rhodesiense*, structure-activity relationship, anti-trypanosomal

## Abstract

In our continued search for natural compounds with activity against *Trypanosoma brucei*, causative agent of human African trypanosomiasis (HAT, “sleeping sickness”), we have investigated extracts from the leaves and bark of the West African *Holarrhena* *africana* (syn. *Holarrhena floribunda*; Apocynaceae). The extracts and their alkaloid-enriched fractions displayed promising in vitro activity against bloodstream forms of *T. brucei rhodesiense* (*Tbr*; East African HAT). Bioactivity-guided chromatographic fractionation of the alkaloid-rich fractions resulted in the isolation of 17 steroid alkaloids, one nitrogen-free steroid and one alkaloid-like non-steroid. Impressive activities (*IC*_50_ in µM) against *Tbr* were recorded for 3β-holaphyllamine (0.40 ± 0.28), 3α-holaphyllamine (0.37 ± 0.16), 3β-dihydroholaphyllamine (0.67 ± 0.03), *N*-methylholaphyllamine (0.08 ± 0.01), conessimine (0.17 ± 0.08), conessine (0.42 ± 0.09), isoconessimine (0.17 ± 0.11) and holarrhesine (0.12 ± 0.08) with selectivity indices ranging from 13 to 302. Based on comparison of the structures of this congeneric series of steroid alkaloids and their activities, structure-activity relationships (SARs) could be established. It was found that a basic amino group at position C-3 of the pregnane or pregn-5-ene steroid nucleus is required for a significant anti-trypanosomal activity. The mono-methylated amino group at C-3 represents an optimum for activity. ∆^5,6^ unsaturation slightly increased the activity while hydrolysis of C-12β ester derivatives led to a loss of activity. An additional amino group at C-20 engaged in a pyrrolidine ring closed towards C-18 significantly increased the selectivity index of the compounds. Our findings provide useful empirical data for further development of steroid alkaloids as a novel class of anti-trypanosomal compounds which represent a promising starting point towards new drugs to combat human African trypanosomiasis.

## 1. Introduction

Human African trypanosomiasis (HAT) is a vector-borne parasitic disease caused by the kinetoplastid parasites, *Trypanosoma brucei rhodesiense* (*Tbr*) and *Trypanosoma brucei gambiense (Tbg)*. The disease ranks 11th, 14th and 4th in the list of neglected tropical diseases (NTDs) for which disability-adjusted life years (DALYs), years of life lost (YLLs) due to mortality and years lived with disability (YLDs) were respectively estimated [1]. Several policy initiatives for prevention and eradication have been developed and funded by World Health Organization WHO and other international organizations [2]. These interventions have been hampered by poor access to available medications, adverse effects associated with some of the medications and incidences of resistant strains of trypanosomes. Moreover, policies geared towards control and eradication of HAT are usually impeded by incomplete and fragmented data on HAT incidence, morbidity and mortality [3] and social stigmatization of the victims in some parts of Sub-Saharan Africa [4]. In view of these obstacles, the search has been on-going to explore additional or alternative remedies from natural products with potential to eradicate HAT. Incidentally, a great number of alkaloids with anti-protozoal activity have been reported and are being explored for antitrypanosomal activity [5,6,7,8,9,10,11,12,13,14,15].

*Holarrhena africana* A. DC. (a synonym of *H*. *floribunda* (G. Don) T. Durand & Schinz) is a tropical tree well distributed in Nigeria and other West African countries. It belongs to the Apocynaceae family which is known to be abundant in alkaloids of various structural classes [16]. Traditionally, the leaves are used in Nigeria to treat convulsion, fever and malaria while the stem bark serves as an antimicrobial, a febrifuge and an antidote for snake venom [17]. Antibacterial and antimalarial [18,19] activities of the chemical constituents of *H*. *africana* have also been reported. A crude aqueous extract of the plant has previously been reported to show interesting preliminary in vitro anti-trypanosomal activity against *Tbr*. A chemically uncharacterized fraction of the aqueous extract has also shown in vivo activity against *T. brucei brucei* [20]. Consequently, we investigated the crude extract and its alkaloid-enriched fraction for anti-trypanosomal activity. Since the alkaloid fraction showed very strong activity, we set out to systematically isolate the alkaloids from *H. africana* and to test them for anti-trypanosomal activity as well as for cytotoxic activity against mammalian cells in order to assess their selectivity and structure-activity relationships (SARs).

## 2. Results and Discussion

### 2.1. Anti-Trypanosomal Activity of Crude Extracts and Alkaloid-Enriched Fractions of Holarrhena africana Leaves and Stem Bark

The crude extracts of the fresh leaves and the dried stem bark of *H. africana* were tested for in vitro activity against *Trypanosoma brucei rhodesiense* (*Tbr*), *Trypanosoma cruzi* (*Tcr*), *Leishmania donovani* (*Ldon*) and *Plasmodium falciparum* (*Pf*) and for cytotoxicity against L6 rat skeletal myoblasts. Since it turned out that activity was selectively observed against *Tbr*, the alkaloidal fraction and column chromatographic fractions were not tested against the other parasites. The results (Table 1) showed that the alkaloid-enriched fraction from the leaf extract had higher activity compared to the crude extract and the polar neutral aqueous fraction (Table 1). These findings are in line with an earlier report [20] of an uncharacterized fraction of *H. africana* leaves with anti-trypanosomal activity, even though the activity reported then was not attributed to the alkaloid content of the plant or to any other chemical characteristic. A similar trend was also observed in the activities of the stem bark extract and its alkaloid fraction. Consequently, both alkaloid fractions were considered potential sources of alkaloids with anti-trypanosomal activity and hence chosen for bioactivity-guided fractionation and isolation of active compounds.

### 2.2. Bio-Activity Guided Fractionation and Isolation of Steroid Alkaloids

**Figure 1:**
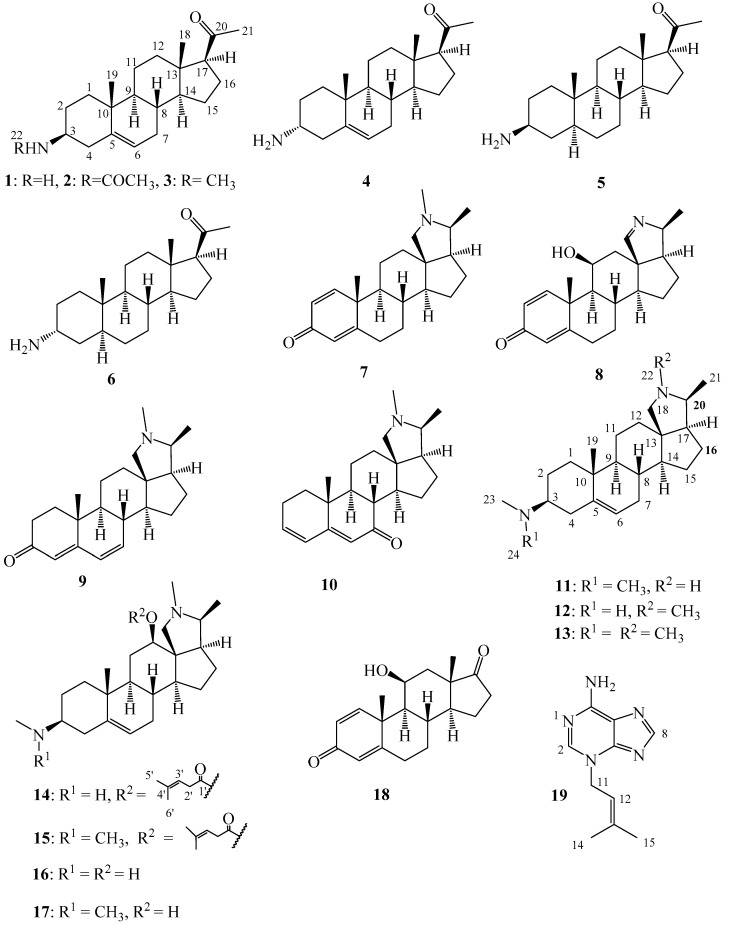
Compounds isolated from the leaves (**1**–**6**, **19**) and stem bark (**7**–**18**) of *Holarrhena africana*.

The alkaloidal fraction of the leaves was fractionated into 16 fractions by column chromatography on silica gel 60. Similarly, the alkaloidal fraction of the stem bark was chromatographed on silica gel column to yield 20 major fractions. All the fractions were analyzed on TLC for the presence or absence of alkaloids using Dragendoff reagent. Positive Dragendoff reaction was obtained in four and two fractions of the leaves and stem bark respectively. The six fractions were subjected to in vitro assay against *Tbr* and higher activities were recorded for three fractions of the leaves and both fractions of the stem bark (Table 1). Further analysis of the fractions by UHPLC/+ESI QTOF MS/MS was carried out and the major alkaloids were partially dereplicated and marked for eventual isolation. Following repeated chromatographic separation and purification, six (**1**–**6**) and eleven (**7**–**17**) major steroid alkaloids were isolated from the leaves and stem bark respectively, in addition to one nitrogen-free steroid (**18**) and a non-steroid alkaloid-like compound (**19)** from the leaves (Figure 1). The compounds were identified as 3β-holaphyllamine (**1**), holaphyllamine acetamide (**2**), 3β-*N*-methylholaphyllamine (**3**), 3α-holaphyllamine (**4**), 3β-dihydroholaphyllamine (**5**), 3α-dihydroholaphyllamine (**6**), holadienine (**7**), holonamine (8), cona-4,6-dienin-3-one (**9**), cona-3,5-dienin-7-one (**10**), conessimine (**11**), isoconessimine (**12**), conessine (**13**), holarrhesine (**14**), holarrhetine (**15**), holarrheline (**16**), holarrhenine (**17**), kurchinin (**18**) and *N^3^*-isopentenyl adenine (**19**) by comparison of their 1D and 2D NMR data and their exact masses from UHPLC/+ESI QTOF MS/MS analysis, which were in agreement with previously reported data [21,22,23,24,25,26,27,28,29,30,31].

### 2.3. In Vitro Anti-Trypanosomal Activity of Isolated Compounds

All the isolated compounds were tested for in vitro anti-trypanosomal activity against *Tbr* and for cytotoxicity against L6 rat skeletal myoblasts. Impressive anti-trypanosomal activities (*IC*_50_, *Tbr* < 1.0 µM) were recorded for compounds **1**, **3**–**5**, **11**–**14**. Compounds **2**, **6** and **15** showed moderate activities (*IC*_50_, 1–5 µM) while compounds **7**–**10**, **16**–**19** were of low activity with *IC*_50_ > 7 µM (Table 2). Interestingly, four steroid alkaloids, **11**–**14** showed very high activity in addition to SI > 100.

### 2.4. Structure-Activity and -Selectivity Relationships

**Figure 2:**
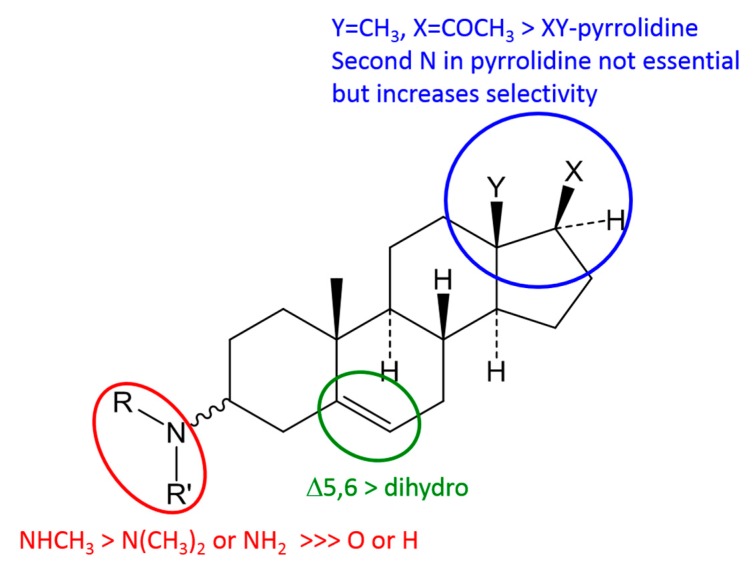
Proposed basic structure-activity relationships for anti-trypanosomal activity. >sign indicates higher activity.

A comparison of all isolated and tested steroidal compounds revealed some structure-activity relationships, as represented in Figure 2.

The major structural requirement for a strong anti-trypanosomal activity against *Tbr* (*IC*_50_ < 1 µM) is a basic amino group at C-3 of the steroid nucleus. Compounds without an amino group in this position such as **7**–**10** and **18** and also the acetamide **2** are considerably less active (*IC*_50_ between 4.8 and 14.9 µM). With respect to substitution of the 3-amino group, monomethylation appears to represent an optimum as observed when comparing monomethylated with unsubstituted (**3** more active than **1**), and monomethylated with dimethylated congeners (**12**, **14**, **16** more active than **13**, **15** and **17**, respectively). Comparing the ∆^5,6^-pregnene derivatives holaphyllamine (**1**) and its 3α-amino analogue (**4**) with their two pregnane congeners **5** and **6**, respectively, shows that the double bond causes a slight increase of activity. Even though **5** with a 3β-amino group is slightly more potent than the α-configured **6**, the effect of stereochemistry at the 3-position appears to be small as indicated by comparison of **1** with **4** which are almost equipotent.

It is very interesting to note that an amino group as part of a pyrrolidine or pyrroline ring connecting C-20 with C-18, as present in compounds **7**–**17**, on its own does not confer high activity (see low activity of **7**–**10**) but rather appears to lead to a slight decrease in antitrypanosomal potency if it occurs together with the 3-amino group (compare **12** with **3**). However, when combined with the C-3 amino function as in **11**–**17**, this structural element leads to a marked decrease of cytotoxicity and increase in selectivity (e.g., SI = 33 in case of **3** and 168 for **12**). This effect is strongest in compound **11** which is not methylated at the pyrrolidine amino group and shows a very favourable SI > 300.

Finally, the introduction of a lipophilic acyloxy substituent at C-12 as in **14** and **15** does not lead to dramatic effects on activity in comparison with the compounds unsubstituted at this position (**12** and **13**, respectively). However, deacylation of such esters leading to a free OH group at C-12 renders compounds **16** and **17** significantly less active.

## 3. Materials and Methods

### 3.1. General Experimental Procedures

Analytical and preparative TLC were performed on pre-coated, silica gel 60 F254 (Merck Chemicals GmbH, Darmstadt, Germany) with various solvent systems consisting of n-hexane, ethyl acetate and methanol saturated with aqueous ammonia as the mobile phase. The plates were visualized under UV-light at 254/360 nm and then sprayed with Dragendoff’s reagent.

### 3.2. Plant Material

The leaves and stem bark of *Holarrhena africana* were collected in March 2015 and April 2016 respectively from a forest reserve in Nsukka (6°51′28′′ N, 7°23′44′′ E), Nigeria. The identities were confirmed by comparison with a voucher specimen (No. 1342003) stored in the herbarium of the Department of Pharmacognosy and Environmental Medicines, University of Nigeria Nsukka. A voucher specimen of this present collection was deposited at the herbarium of the Institute of Pharmaceutical Biology and Phytochemistry, University of Münster (voucher No. TS-HA-01). The stem bark was air dried at room temperature and ground to 1 mm mesh size. The fresh leaves were washed with distilled water and allowed to drain completely.

### 3.3. Extraction and Preparation of Alkaloid-Enriched Fractions

*Fresh leaves:* A 2 kg fresh weight of the leaves was extracted as previously described [20]. The extract was filtered and lyophilized for 18 h to dry weight of 28.6 g. The crude extract was re-dissolved in 10% *v*/*v* MeOH/distilled water (in portions of 1.0 g to 50 mL) and basified with 1% *v*/*v* NH_4_OH to pH 12. The mixture was partitioned five times successively with CH_2_Cl_2_ (in portions of 1:1 ratio) in a separatory funnel. The combined lower phase was evaporated completely to yield 8.4 g (28.6% of crude extract) of alkaloid-enriched (AFL) fraction, 5.2 g of precipitate and 14.7 g polar aqueous fraction.

*Stem bark:* The dry powdered stem bark (500 g, in portions of 5 × 100 g) was extracted exhaustively for 12 h in a Soxhlet apparatus with a total of 4.0 L dichloromethane. The solvent was evaporated in vacuo at 40 °C. The extract yield was 58.6 g. The resulting crude extract (50 g) was re-dissolved in several portions of 10% *v*/*v* MeOH/water in a ratio of 50 mL/1 g extract, basified as described above and subsequently partitioned successively with CH_2_Cl_2_ in a separatory funnel to yield 14.5 g of alkaloid-enriched fraction (AFS), 8.1 g of precipitate, and 26.6 g polar aqueous fraction.

The crude extracts and alkaloid-enriched fractions were tested for anti-trypanosomal activity and cytotoxicity (see Table 1).

### 3.4. Isolation of Steroid Alkaloids

#### 3.4.1. Isolation of Alkaloids from the Leaf Extract

The major part (8 g) of the AFL was subjected to column chromatographic (CC) separation on 560 g silica gel (E. Merck, type-60, 70–230 mesh) and eluted with gradients of (50% n-hexane to 100% EtOAc) *n*-hexane–EtOAc mixture *v*/*v*, each gradient saturated with NH_4_OH (25% *v*/*v*). The mobile phase solvent was constituted as follows: 2 L each of 1:1, 3:7 and 1:9, followed by 6 L EtOAc and finally EtOAc:MeOH (9.9:0.1). The flow rate was maintained at 1 mL/min. The eluates were analyzed at pre-determined intervals and based on their TLC profiles and positive reaction to Dragendoff’s spraying reagent, representative tubes (127–305) were collected and pooled to yield four alkaloid-containing sub-fractions, HA11 (1250 mg), HA12 (750 mg), HA13 (1680 mg) and HA14 (480 mg). These sub-fractions were tested for anti-trypanosomal activity (Table 1). Compound **19** (25.8 mg) was obtained as pure crystal by repeatedly washing sub-fraction HA11 with n-hexane. HA12 (700 mg) was re-chromatographed on silica gel 60 and eluted isocratically with NH_4_OH saturated-EtOAc and at flow rate of 0.5 mL/min. Compound **2** (10.0 mg) crystallized as white flaky substance while compound **1** (6.8 mg) was isolated after further purification on sephadex LH20 gel eluted with MeOH:H_2_O, 9:1. HA13 (1600 mg) was further separated by column chromatography on 80 g basic alumina with EtOAc:MeOH (9:1) as mobile phase to yield three sub-fractions (HA13a, HA13b, and HA13c). Compound **3** (52 mg) was isolated as a sticky white creamy solid from sub-fraction HA13b (220 mg) by repeatedly washing with *n*-hexane:EtOAc (9:1). HA13a (120 mg) was further chromatographed on a pre-coated preparative TLC plates (GF-254, 20 × 20 cm, E. Merck, 0.20 mm thickness, Darmstadt, Germany) developed in pre-equilibrated TLC tank containing EtOAc:MeOH (9.5:0.5) and saturated with NH_4_OH. The plates were scrapped according to bands identified after spraying a 2 × 20 cm cut-off segment of the plate with Dragendoff reagent, re-extracted and washed with EtOAc to furnish compounds **4** (2.5 mg; hRf 36) and **6** (3.8 mg; hRf 23). HA14 and HA13c (80 mg) were combined and further separated by CC on Sephadex LH-20 using MeOH:H_2_O (8:2) as eluent. The alkaloid-positive eluates were pooled and further purified on preparative TLC (silica gel 60, in NH_4_OH-saturated EtOAc) to furnish compound **5** (4.1 mg, hRf 22) as a creamy solid.

#### 3.4.2. Isolation of Alkaloids from the Stem Bark Extract

Similarly, a major portion (12.0 g) of AFS was subjected to CC on 900 g silica gel 60. The column was eluted with mobile phase solvents of increasing polarity, as described above. The eluates were analyzed by TLC and, based on a positive reaction to Dragendoff reagent, it was observed that most of the alkaloidal bands were overlapping. Therefore, alkaloid positive eluates were pooled in two fractions, HF8 (eluates 86–232; 3.85 g) and HF9 (eluates 233–480; 5.33 g). These fractions were also tested for anti-trypanosomal activity (Table 1). HF8 (3.8 g) was re-chromatographed over 270 g silica gel 60. The column was eluted with 5.0 L of EtOAc (100%) saturated with NH_4_OH at a flow rate of 0.8 mL/min. 205 eluates were collected and representative fractions pooled into five sub-fractions (HF8a-HF8e) based on the positive reaction to the Dragendoff reagent. HF8a (80 mg) was purified on basic alumina with EtOAc:MeOH (9.5:0.5) as eluent to yield compound **10** (9.6 mg). HF8b (108 mg) and HF8c (228 mg) were combined and re-chromatographed on silica gel 60 and eluted with *n*-hexane:EtOAc (2:8) saturated with NH_4_OH to yield compounds **9** and **7** as a mixture. The mixture was separated on pre-coated preparative TLC silica plates in EtOAc (100%) saturated with NH_4_OH to yield 5.6 mg of **9** (hRf 65) and 4.2 mg of **7** (hRf 60) respectively. HF8d (210 mg) was chromatographed on silica gel 60 and eluted subsequently with EtOAc (100%) to yield crystals of **18** (3.8 mg) and then with EtOAc–NH_4_OH to yield **8** (7.9 mg) as white solid. HF8e (675 mg) was further separated on silica gel 60 (50 g) with EtOAc (100%) saturated with NH_4_OH, flow rate 0.5 mL/min to yield two sticky sub-fractions which were further purified on pre-coated preparative TLC with NH_4_OH-saturated EtOAc as mobile phase to furnish **15** (16.7 mg, hRf 55) and **14** (15.6 mg; hRf 48). HF9 (5.0 g) was re-chromatographed on silica gel 60 (300 g) and eluted with 2.5 L EtOAc (100%) followed by 3.0 L EtOAc:MeOH (9.8:0.2) both saturated with NH_4_OH to yield three sub-fractions, HF9a (860 mg), HF9b (510 mg) and HF9c (1050 mg). HF9a yielded further **14** and **15** in a mixture. HF9b was purified by CC on basic alumina with EtOAc:MeOH (8:2) as mobile phase to yield **13** (3.8 mg) and **12** (4.1 mg). HF9c yielded **17** (8.8 mg; hRf 58), **11** (10.8 mg; hRf 50) and **16** (5.2 mg; hRf 39) after further separation by CC on silica with EtOAc:MeOH (9:1) satutated with NH_4_OH as mobile phase and subsequent purification by preparative TLC (silica 60, EtOAc:MeOH (8:2) saturated with NH_4_OH as mobile phase).

#### 3.4.3. Preparation of Compounds **16** and **17** by Alkaline Hydrolysis of the Esters **14** and **15**

To increase the yields and further confirm **16** and **17**, a simple, efficient and reliable ester hydrolysis, without racemization or other undesirable side reaction protocol [32] was adopted. 10 mg each of compound **14** and **15** was respectively hydrolyzed in CH_2_Cl_2_ using 0.5 N NaOH (dissolved in MeOH) at room temperature for 2 h; CH_2_Cl_2_:MeOH (9:1). The reaction mixtures were stirred vigorously and monitored by TLC until completion and purified by preparative TLC as previously described. The yield in each case was >70%.

### 3.5. Structural Characterization of Isolated Compounds

NMR spectra (^1^H, ^13^C, ^1^H/^1^H COSY, ^1^H/^1^H NOESY, ^1^H/^13^C HSQC, and ^1^H/^13^C HMBC) were recorded on Agilent DD2 400 or 600 MHz spectrometers at 25 °C in CDCl_3_ or CD_3_OD. Spectra were respectively referenced to the CDCl_3_ solvent signals of ^1^H; 7.260 ppm and ^13^C; 77.000 ppm or CD_3_OD solvent signals of ^1^H; 3.310 ppm and ^13^C; 49.000 ppm and processed with MestRENOVA v. 11 (Mestrelab Research, Chemistry Software Solutions, Santiago de Compostela, Spain) software.

### 3.6. Spectral Data of Isolated Compounds

3β-Holaphyllamine **1**: hRf (silica gel 60 tlc, EtOAc:MeOH, 9.5:0.5-saturated aq. NH_3_), 40.0; ^1^H-NMR (600 MHz CDCl_3_; δ (ppm), intensity, mult., *J* (Hz)): 5.32 (1H, *dt*, 5.3, 1.9, H-6), 2.60 (1H, *tt*, 4.1, 11.5, H-3), 2.53 (1H, *t*, 9.2, H-17), 2.17 (1H, *m*, H-16β), 2.15 (1H, *m*, H-4β), 2.12 (3H, *s*, H-21), 2.06 (1H, *m*, H-12α), 2.05 (1H, *m*, H-4α), 1.98 (1H, *dq*, 2.7, 5.2, H-7α), 1.85 (1H, *dt*, 12.7, 3.2, H-1β), 1.83 (1H, *dt*, 12.7, 3.2, H-1α), 1.72 (1H, *m*, H-2β), 1.70 (1H, *m*, H-2α), 1.69 (1H, *m*, H-15β), 1.66 (1H, *m*, H-16α), 1.62 (1H, *m*, H-11α), 1.57 (1H, *m*, H-7β), 1.47 (1H, *m*, H-8), 1.45 (1H, *m*, H-11β), 1.45 (1H, *m*, H-12β), 1.23 (1H, *m*, H-15α), 1.16 (1H, *m*, H-14), 1.0 (1H, *m*, H-9), 0.99 (3H, *s*, H-19), 0.63 (3H, *s*, H-18). MS (*m*/*z*): 316.2845 [M + H]^+^; calculated for C_21_H_34_NO^+^: 316.2640.

3β-Holaphyllamine acetamide **2**: hRf (silica gel 60 tlc, EtOAc:MeOH, 9.5:0.5-saturated aq. NH_3_), 74.0; ^1^H-NMR (600 MHz CDCl_3_; δ (ppm), intensity, mult., *J* (Hz)): 5.36 (1H, *dt*, 5.0, 2.2, H-6), 3.70 (1H, *tdd*, 14.8, 8.2, 4.5, H-3), 2.54 (1H, *t*, 8.9, H-17), 2.31 (2H, *ddd*, 13.2, 4.7, 2.2, H-4), 2.16 (1H, *m*, H-15α), 2.11 (3H, *s*, H-21), 2.05 (1H, *m*, H-12α), 1.98 (1H, *m*, H-7α), 1.95 (3H, *s*, H-23), 1.86 (1H, *m*, H-2α), 1.85 (1H, *m*, H-1α), 1.68 (1H, *m*, H-16α), 1.65 (1H, *m*, H-15β), 1.61 (1H, *m*, H-11α), 1.58 (1H, *m*, H-7β), 1.46 (1H, *m*, H-8), 1.45 (1H, *m*, H-12β), 1.45 (1H, *m*, H-11β), 1.35 (1H, *m*, H-2β), 1.20 (1H, *m*, H-16β), 1.17 (1H, *m*, H-1β), 1.15 (1H, *m*, H-14), 1.01 (1H, *m*, H-9), 0.98 (3H, *s*, H-19), 0.62 (3H, *s*, H-18). MS (*m*/*z*): 358.3000 [M + H]^+^; calculated for C_23_H_36_NO_2_^+^: 358.2746.

3β-*N*-Methylholaphyllamine **3**: hRf (silica gel 60 tlc, EtOAc:MeOH, 9.5:0.5-saturated aq. NH_3_), 54.0; ^1^H-NMR (600 MHz CDCl_3_; δ (ppm), intensity, mult., *J* (Hz)): 5.40 (1H, *dt*, 5.5, 2.0, H-6), 2.76 (1H, *tt*, 4.1, 11.7, H-3), 2.62 (3H, *s*, H-22), 2.54 (1H, *dd*, 13.3, 2.3, H-4α), 2.51 (1H, *dd*, 2.4, 5.0, H-17), 2.47 (1H, *ddd*, 13.4, 4.4, 2.3, H-4β), 2.16 (1H, *m*, H-15α), 2.11 (3H, *s*, H-21), 2.04, (1H, *m*, H-2α), 2.03 (1H, *m*, H-12α), 1.99 (1H, *m*, H-7α), 1.92 (1H, *m*, H-1α), 1.82, *qd*, 12.8, 3.6, H-2β), 1.66 (1H, *m*, H-16α), 1.64 (1H, *m*, H-15β), 1.57 (1H, *m*, H-7β), 1.57 (1H, *m*, H-11α), 1.46 (1H, *m*, H-8), 1.44 (1H, *m*, H-12β), 1.43 (1H, *m*, H-11β), 1.20 (1H, *m*, H-16β), 1.14 (1H, *m*, H-14), 1.08 (1H, *m*, H-1β), 1.01 (3H, *s*, H-19), 0.98 (1H, *m*, H-9), 0.60 (3H, *s*, H-18). MS (*m*/*z*): 330.2819 [M + H]^+^; calculated for C_22_H_36_NO^+^: 330.2797.

3α-Holaphyllamine **4**: hRf (silica gel 60 tlc, EtOAc:MeOH, 9.5:0.5-saturated aq. NH_3_), 36.0; ^1^H-NMR (600 MHz CDCl_3_; δ (ppm), intensity, mult., *J* (Hz)): 5.33 (1H, *dt*, 5.3, 2.2, H-6), 3.16 (1H, *br. s*, H-3), 2.56 (1H, *m*, H-4β), 2.51 (1H, *t*, 9.0, H-17), 2.15 (1H, *m*, H-16β), 2.10 (3H, *s*, H-21), 2.02 (1H, *m*, H-12β), 1.96 (1H, *m*, H-7α), 1.88 (1H, *dt*, 14.9, 2.7, H-4α), 1.78 (1H, *dt*, 14.1, 3.7, H-2β), 1.65 (1H, *m*, H-15β), 1.62 (1H, *m*, H-16α), 1.60 (1H, *m*, H-11β), 1.60 (1H, *m*, H-1α), 1.58 (1H, *m*, H-7β), 1.48 (1H, *m*, H-2α), 1.44 (1H, *m*, H-8), 1.42 (1H, *m*, H-11α), 1.42 (1H, *m*, H-12α), 1.36 (1H, *m*, H-1β), 1.18 (1H, *m*, H-15α), 1.13 (1H, *m*, H-14), 1.07 (1H, *m*, H-9), 0.97 (3H, *s*, H-19), 0.60 (3H, *s*, H-18). MS (*m*/*z*): 316.2827 [M + H]^+^; calculated for C_21_H_34_NO^+^: 316.2640.

3β-Dihydroholaphyllamine **5**: hRf (silica gel 60 tlc, EtOAc:MeOH, 9.5:0.5-saturated aq. NH_3_), 42.0; ^1^H-NMR (600 MHz CD_3_OD; δ (ppm), intensity, mult., *J* (Hz)): 3.05 (1H, *tt*, 11.9, 4.5, H-3), 2.62 (1H, *t*, 9.1, H-17), 2.12 (1H, *m*, H-16α), 2.10 (3H, *s*, H-21), 2.08, *m*, H-12α), 2.03 (1H, *dt*, 12.1, 3.4, H-12β), 1.86 (1H, *m*, H-2β), 1.84 (1H, *m*, H-2α), 1.82 (1H, *m*, H-1α), 1.74 (1H, *m*, H-16β), 1.60 (1H, *m*, H-4α), 1.42 (2H, *m*, H-5), 1.40 (1H, *m*, H-4β), 1.36 (1H, *m*, H-11β), 1.33 (1H, *m*, H-11α), 1.32 (1H, *m*, H-6α), 1.30 (1H, *m*, H-6β), 1.22 (1H, *m*, H-8), 1.21 (1H, *m*, H-14), 1.20 (2H, *m*, H-15), 1.08 (1H, *m*, H-1β), 0.97 (2H, *m*, H-7), 0.86 (3H, *s*, H-19), 0.78 (1H, *ddd*, 12.3, 10.7, 4.1, H-9), 0.60 (3H, *s*, H-18). MS (*m*/*z*): 318.2809 [M + H]^+^; calculated for C_21_H_36_NO^+^: 318.2797.

3α-Dihydroholaphyllamine **6**: hRf (silica gel 60 tlc, EtOAc:MeOH, 9.5:0.5-saturated aq. NH_3_), 23.0; ^1^H-NMR (600 MHz CDCl_3_; δ (ppm), intensity, mult., *J* (Hz)): 3.21 (1H, *br s*, H-3), 2.52 (1H, *t*, 9.0, H-17), 2.14 (1H, *m*, H-16β), 2.11 (3H, *s*, H-21), 2.01 (1H, *dt*, 12.2, 3.4, H-12β), 1.74 (1H, *m*, H-2α), 1.68 (1H, *m*, H-1α), 1.66 (1H, *m*, H-15α), 1.64 (1H, *m*, H-16α), 1.62 (1H, *m*, H-11α), 1.55 (1H, *dd*, 3.8, 13.5, H-4α), 1.48 (1H, *m*, H-5), 1.46 (1H, *m*, H-6α), 1.45 (1H, *m*, H-7α), 1.44 (1H, *m*, H-6β), 1.39 (1H, *m*, H-12α), 1.36 (1H, *m*, H-8), 1.28 (1H, *m*, H-7β), 1.26 (1H, *m*, H-11β), 1.21 (1H, *m*, H-4β), 1.20 (1H, *m*, H-2β), 1.17 (1H, *m*, H-15β), 1.16 (1H, *m*, H-14), 0.95 (1H, *m*, H-1β), 0.80 (1H, *td*, 3.2, 5.4, H-9), 0.78 (3H, *s*, H-19), 0.60 (3H, *s*, H-18). MS (*m*/*z*): 318.2788 [M + H]^+^; calculated for C_21_H_36_NO^+^: 318.2797.

Holadienine **7**: hRf (silica gel 60 tlc, EtOAc:MeOH, 9.9:0.1-saturated aq. NH_3_), 81.0; ^1^H-NMR (600 MHz CDCl_3_; δ (ppm), intensity, mult., *J* (Hz)): 7.04 (1H, *d*, 10.1, H-1), 6.22 (1H, *dd*, 10.1, 1.9, H-2), 6.07 (1H, *t*, 1.6, H-4), 3.09 (1H, *br s*, H-18α), 2.46 (1H, *ddd*, 13.4, 5.1, 1.5, H-6α), 2.45 (1H, *dd*, 5.1, 1.4, H-17), 2.32 (1H, *ddd*, 13.3, 4.2, 2.6, H-6β), 2.25 (3H, *s*, H-22), 2.04 (1H, *m*, H-7α), 1.95 (1H, *br s*, H-18β), 1.85 (1H, *m*, H-20), 1.83 (1H, *m*, H-11α), 1.83 (1H, *m*, H-12α), 1.76 (1H, *dd*, H-16α), 1.64 (2H, *qd*, H-15), 1.50 (1H, *qd*, 10.9, 3.8, H-8), 1.43 (1H, *dd*, H-16β), 1.35 (1H, *m*, H-12β), 1.35 (1H, *m*, H-11β), 1.18 (3H, *s*, H-19), 1.12 (1H, *m*, H-7β), 1.12 (1H, *m*, H-14), 1.08, 3H, *d*, H-21), 1.07 (1H, m, H-9). MS (*m*/*z*): 326.3834 [M + H]^+^; calculated for C_22_H_32_NO^+^: 326.2484.

Holonamine **8**: hRf (silica gel 60 tlc, EtOAc:MeOH, 9.9:0.1-saturated aq. NH_3_), 74.0; ^1^H-NMR (600 MHz CDCl_3_; δ (ppm), intensity, mult., *J* (Hz)): 7.86 (1H, *d*, 10.3, H-4), 7.48 (1H, *d*, 3.0, H-18), 6.15 (1H, *dd*, 10.3, 2.0, H-2), 6.10 (1H, *t*, 1.6, H-1), 4.05–4.10 (2H, *m*, H-11, H-17),), 2.51 (1H, *tdd*, 13.5, 5.1, 1.5, H-6α), 2.39 (1H, *ddd*, 13.2, 4.4, 2.5, H-6β), 2.19 (1H, *dd*, 12.2, 4.8, H-12α), 2.10 (1H, *m*, H-20), 2.06 (1H, *m*, H-7α), 1.91 (1H, *qd*, 11.4, 3.8, H-8), 1.78 (1H, *ddd*, 14.0, 8.2, 2.8, H-15α), 1.68 (1H, *m*, H-16α), 1.63 (1H, *dd*, 12.2, 4.8, H-12β), 1.45 (1H, *ddd*, 14.2, 8.5, 3.0, H-15β), 1.38 (1H, *td*, 11.5, 5.3, H-14), 1.36 (3H, *s*, H-19), 1.35 (3H, *d*, H-21), 1.32 (1H, *m*, H-9), 1.15 (1H, *ddd*, 12.8, 5.0, 3.8, H-7β), 0,83 (1H, *m*, H-16β). MS (*m*/*z*): 326.2000 [M + H]^+^; calculated for C_21_H_28_NO_2_^+^: 326.2120.

Cona-4,6-dienin-3-one **9**: hRf (silica gel 60 tlc, EtOAc:MeOH, 9.9:0.1-saturated aq. NH_3_), 84.0; ^1^H-NMR (600 MHz CDCl_3_; δ (ppm), intensity, mult., *J* (Hz)): 6.13 (1H, *m*, H-6), 6.02 (1H, *m*, H-7), 5.64 (1H, *s*, H-4), 2.96 (1H, *d*, 10.4, H-18β), 2.33 (1H, *m*, H-20), 2.32 (2H, *m*, H-2), 2.19 (1H, *m*, H-1α), 2.16 (3H, *s*, H-22), 1.86 (1H, *m*, H-1β), 1.85 (1H, *d*, H-18α), 1.78 (1H, *dt*, 10.7, 3.7, H-17), 1.74 (1H, *dt*, 12.2, 3.4, H-12α), 1.64 (2H, *ddd*, 17.1, 10.3, 4.2, H-15), 1.58 (1H, *m*, H-16α), 1.57 (1H, *m*, H-11α), 1.35 (1H, *dt*, 10.8, 3.0, H-8), 1.28 (1H, *td*, 12.8, 3.8, H-12β), 1.14 (1H, *dq*, 11.0, 2.6, H-16β), 1.08 (1H, *m*, H-14), 1.04 (1H, *m*, H-11β), 1.05 (3H, *s*, H-19), 0.98 (3H, *d*, 6.4, H-21), 0.87 (1H, *ddd*, 12.3, 10.5, 3.6, H-9). MS (*m*/*z*): 326.3869 [M + H]^+^; calculated for C_22_H_32_NO^+^: 326.2484.

Cona-3,5-dienin-7-one **10**: hRf (silica gel 60 tlc, EtOAc:MeOH, 9.9:0.1-saturated aq. NH_3_), 85.0; ^1^H-NMR (600 MHz CDCl_3_; δ (ppm), intensity, mult., *J* (Hz)): 6.14 (1H, *ddd*, 9.5, 5.4, 2.6, H-3), 6.06 (1H, *dd*, 9.7, 2.4, H-4), 5.58 (1H, *s*, H-6), 2.95 (1H, *d*, 10.3, H-18α), 2.47 (1H, *ddd*, 13.0, 6.5, 4.1, H-16α), 2.38 (1H, *dd*, 6.5, 4.6, H-20), 2.25 (2H, *m*, H-2), 2.22 (1H, *m*, H-8), 2.18 (3H, *s*, H-22), 1.86 (1H, *m*, H-1β), 1.85 (1H, *m*, H-18β), 1.78 (1H, *m*, H-12β), 1.74 (1H, *m*, H-17), 1.72 (1H, *m*, H-11β), 1.70 (1H, *m*, H-15β), 1.61 (1H, *m*, H-9), 1.42 (1H, *m*, H-14), 1.41 (1H, *m*, H-15α), 1.39 (1H, *m*, H-16β), 1.31 (1H, *m*, H-1α), 1.30 (1H, *m*, H-12α), 1.23 (1H, *m*, H-11α), 1.03 (3H, *s*, H-19), 1.02 (3H, *d*, 6.3, H-21). MS (*m*/*z*): 326.3871 [M + H]^+^; calculated for C_22_H_32_NO^+^: 326.2484.

Conessimine **11**: hRf (silica gel 60 tlc, EtOAc:MeOH, 9.9:0.1-saturated aq. NH_3_), 47.0; ^1^H-NMR (600 MHz CDCl_3_; δ (ppm), intensity, mult., *J* (Hz)): 5.35 (1H, *dt*, 5.1, 1.8, H-6), 3.19 (1H, *qd*, 6.5, 4.7, H-20), 2.79 (1H, *d*, 12.1, H-18β), 2.52 (1H, *d*, 12.1, H-18α), 2.27 (6H, *s*, H-22, H-23), 2.19 (2H, *m*, H-4), 2.10 (1H, *m*, H-3), 2.08 (1H, *m*, H-7α), 2.05 (1H, *m*, H-7β), 1.94 (1H, *m*, H-1α), 1.89 (1H, *m*, H-17), 1.88 (1H, *m*, H-12α), 1.78 (1H, *m*, H-2α), 1.69 (1H, *m*, H-16α), 1.69 (1H, *m*, H-11α), 1.64 (1H, *m*, H-15α), 1.51 (1H, *m*, H-15β), 1.46 (1H, *m*, H-2β), 1.41 (1H, *m*, H-12β), 1.32 (1H, *m*, H-8), 1.31 (1H, *m*, H-14), 1.23 (1H, *m*, H-11β), 1.16 (1H, *m*, H-16β), 1.12 (3H, *d*, 6.6, H-21), 1.08 (1H, *m*, H-1β), 0.99 (1H, *m*, H-9), 0.93 (3H, *s*, H-19). MS (*m*/*z*): 343.4082 [M + H]^+^; calculated for C_23_H_39_N_2_^+^: 343.3113.

Isoconessimine **12**: hRf (silica gel 60 tlc, EtOAc:MeOH, 9.9:0.1-saturated aq. NH_3_), 55.0; ^1^H-NMR (600 MHz CDCl_3_; δ (ppm), intensity, mult., *J* (Hz)): 5.37 (1H, *td*, 5.0, 2.2, H-6), 3.30 (1H, *d*, 11.3, H-18α), 2.73 (1H, *m*, H-20), 2.59 (1H, *m*, H-3), 2.54 (3H, *s*, H-23), 2.42 (3H, *s*, H-22), 2.35 (2H, *m*, H-4), 2.12 (1H, *m*, H-18β), 2.08 (1H, *td*, 5.4, 2.0, H-7α), 1.97 (1H, *m*, H-17), 1.92 (1H, *m*, H-2α), 1.89 (1H, *m*, H-1α), 1.82 (1H, *m*, H-12β), 1.82 (1H, *m*, H-15α), 1.68 (1H, *m*, H-16α), 1.67 (1H, *m*, H-11α), 1.61 (1H, *m*, H-2β), 1.60 (1H, *m*, H-7β), 1.51 (1H, *dt*, 10.7, 2.8, H-15β), 1.40 (1H, *dd*, 11.3, 4.1, H-12α), 1.34 (1H, *m*, H-8), 1.31 (1H, *m*, H-16β), 1.19 (3H, *d*, 6.5, H-21), 1.17 (1H, *m*, H-11β), 1.16 (1H, *m*, H-14), 1.08 (1H, *m*, H-1β), 0.97 (1H, *m*, H-9), 0.94 (3H, *s*, H-19). MS (*m*/*z*): 343.4055 [M + H]; calculated for C_23_H_39_N_2_^+^: 343.3113

Conessine **13**: hRf (silica gel 60 tlc, EtOAc:MeOH, 9.9:0.1-saturated aq. NH_3_), 70.0; ^1^H-NMR (600 MHz CDCl_3_; δ (ppm), intensity, mult., *J* (Hz)): 5.32 (1H, *m*, H-6), 2.97 (1H, *d*, 10.3, H-18α), 2.34 (1H, *m*, H-20), 2.27 (6H, *s*, H-23,24), 2.18 (3H, *s*, H-22), 2.18 (2H, *m*, H-4), 2.09 (1H, *m*, H-3), 2.06 (1H, *m*, H-7α), 1.87 (1H, *m*, H-1α), 1.85 (1H, *m*, H-18β), 1.82 (1H, *m*, H-17), 1.76 (1H, *m*, H-12α), 1.73 (1H, *m*, H-2α), 1.68 (1H, *m*, H-15α), 1.63 (1H, *m*, H-11α), 1.61 (1H, *m*, H-11β), 1.59 (2H, *m*, H-16), 1.58 (1H, *m*, H-7β), 1.41 (1H, *m*, H-2β), 1.39 (1H, *m*, H-15β), 1.36 (1H, *m*, H-8), 1.33 (1H, *m*, H-12β), 1.10 (1H, *m*, H-14), 1.04 (1H, *m*, H-1β), 1.01 (3H, *d*, 6.3, H-21), 0.93 (1H, *m*, H-9), 0.90 (3H, *s*, H-19). MS (*m*/*z*): 357.4072 [M + H]^+^; calculated for C_24_H_41_N_2_^+^: 357.3270

Holarrhesine **14**: hRf (silica gel 60 tlc, EtOAc:MeOH, 9.9:0.1-saturated aq. NH_3_), 69.0; ^1^H-NMR (600 MHz CDCl_3_; δ (ppm), intensity, mult., *J* (Hz)): 5.34 (1H, m, H-6), 5.31 (1H, *ddt*, 7.3, 2.9, 1.4, H-3′), 4.89 (1H, *dd*, 11.3, 4.2, H-12), 3.02 (2H, *dp*, 7.3, 1.0, H-2′), 2.84 (1H, *d*, 10.1, H-18β), 2.41 (3H, *s*, H-23), 2.34 (1H, *d*, 10.2, H-18α), 2.32 (1H, *m*, H-20), 2.27 (1H, *m*, H-3), 2.24 (1H, *dd*, 4.3, 2.1, H-4α), 2.22 (3H, *s*, H-22), 2.10 (1H, *m*, H-17), 2.08 (1H, *m*, H-7α), 2.00 (1H, *m*, H-4β), 1.78 (1H, *m*, H-11α), 1.78 (1H, *m*, H-1α), 1.74 (3H, *s*, H-5′), 1.71 (1H, *m*, H-15β), 1.65 (2H, *m*, H-16), 1.65 (3H, *s*, H-6′), 1.58 (1H, *m*, H-7β), 1.39 (1H, *m*, H-15α), 1.38 (1H, *m*, H-8), 1.27 (1H, *m*, H-2β), 1.25 (1H, *m*, H-2α), 1.20 (1H, *m*, H-11β), 1.20 (1H, *m*, H-14), 1.12 (1H, *m*, H-9), 1.08 (1H, *m*, H-1β), 0.98 (3H, *d*, 6.4, H-21), 0.93 (3H, *s*, H-19). MS (*m*/*z*): 455.5514 [M + H]; calculated for C_29_H_47_N_2_O_2_^+^: 455.3638.

Holarrhetine **15**: hRf (silica gel 60 tlc, EtOAc:MeOH, 9.9:0.1-saturated aq. NH_3_), 73.0; ^1^H-NMR (600 MHz CDCl_3_; δ (ppm), intensity, mult., *J* (Hz)): 5.32 (1H, *dt*, 4.1, 1.6, H-6), 5.30 (1H, *ddt*, 7.1, 2.7, 1.4, H-3′), 4.89 (1H, *dd*, 11.1, 4.2, H-12), 3.01 (2H, *dp*, 7.5, 1.0, H-2′), 2.84 (1H, *d*, 10.2, H-18α), 2.34 (1H, *d*, 10.4, H-18β), 2.32 (1H, *m*, H-20), 2.28 (6H, *s*, H-23, H-24), 2.22 (3H, *s*, H-22), 2.16 (2H, *m*, H-4), 2.10 (1H, *m*, H-17), 2.08 (1H, *m*, H-3), 2.06 (1H, *m*, H-7α), 1.82 (1H, *m*, H-1α), 1.78 (1H, *dd*, 10.0, 4.9, H-11α), 1.72 (1H, *m*, H-15β), 1.72 (1H, *m*, H-2α), 1.70 (3H, *s*, H-5′), 1.64 (3H, *s*, H-6′), 1.64 (1H, *m*, H-16α), 1.58 (1H, *m*, H-7β), 1.38 (1H, *m*, H-2β), 1.38 (1H, *m*, H-15α), 1.34 (1H, *dd*, H-8), 1.25 (1H, *m*, H-16β), 1.20 (1H, *dd*, H-14), 1.16 (1H, *dd*, 6.6, 3.1, H-11β), 1.12 (1H, *dd*, 3.6, 2.2, H-9), 1.05 (1H, *m*, H-1β), 0.98 (3H, *d*, 6.4, H-21), 0.92 (3H, s, H-19). MS (*m*/*z*): 469.5725 [M + H]^+^; calculated for C_30_H_49_N_2_O_2_^+^: 469.3794.

Holarrheline **16**: hRf (silica gel 60 tlc, EtOAc:MeOH, 9.9:0.1-saturated aq. NH_3_), 31.0; ^1^H-NMR (600 MHz CD_3_OD; δ (ppm), intensity, mult., *J* (Hz)): 5.35 (1H, *dt*, 5.4, 1.8, H-6), 3.58 (1H, *dd*, 11.0, 4.2, H-12), 2.77 (1H, *d*, 10.5, H-18α), 2.67 (1H, *qd*, 6.4, 4.9, H-20), 2.41 (1H, *d*, 10.5, H-18β), 2.36 (3H, *s*, H-23), 2.28 (1H, *m*, H-4α), 2.27 (1H, *m*, H-3), 2.25 (3H, *s*, H-22), 2.19 (1H, *ddd*, 10.8, 4.9, 3.0, H-17), 2.09 (1H, *td*, 5.3, 2.6, H-7α), 2.05 (1H, *m*, H-4β), 1.88 (1H, *m*, H-1α), 1.82 (1H, *m*, H-2β), 1.73 (1H, *m*, H-15β), 1.73 (1H, *m*, H-11α), 1.64 (1H, *m*, H-16β), 1.60 (1H, *m*, H-7β), 1.44 (1H, *m*, H-15α), 1.34 (1H, *m*, H-2α), 1.33 (1H, *m*, H-8), 1.32 (1H, *m*, H-16α), 1.15 (1H, *td*, 6.3, 1.5, H-14), 1.13 (1H, *m*, H-11β), 1.10 (1H, *m*, H-1β), 1.08 (1H, *m*, H-9), 1.04 (3H, *d*, 6.5, H-21), 0.95 (3H, *s*, H-19). MS (*m*/*z*): 359.4531 [M + H]^+^; calculated for C_23_H_39_N_2_O^+^: 359.3062.

Holarrhenine **17**: hRf (silica gel 60 tlc, EtOAc:MeOH, 9.9:0.1-saturated aq. NH_3_), 55.0; ^1^H-NMR (600 MHz CD_3_OD; δ (ppm), intensity, mult., *J* (Hz)): 5.35 (1H, dt, 5.3, 1.9, H-6), 3.58 (1H, dd, 11.1, 4.2, H-12), 2.78 (1H, d, 10.5, H-18β), 2.68 (1H, *m*, H-20), 2.41 (1H, *d*, 10.6, H-18α), 2.27 (6H, *s*, H-23, H-24), 2.25 (3H, *s*, H-22), 2.23 (1H, *m*, H-4α), 2.18 (1H, *m*, H-4β), 2.18 (1H, *m*, H-14), 2.10 (1H, *m*, H-3), 2.07 (1H, *m*, H-7α), 1.90 (1H, *m*, H-1β), 1.78 (2H, *m*, H-2), 1.73 (1H, *m*, H-11α), 1.72 (1H, *m*, H-15α), 1.64 (1H, *m*, H-16α), 1.58 (1H, *m*, H-7β), 1.46 (1H, *m*, H-15β), 1.32 (1H, *m*, H-8), 1.30 (1H, *m*, H-16β), 1.15 (1H, *m*, H-17), 1.11 (1H, *m*, H-11β), 1.07 (1H, *m*, H-1α), 1.06 (1H, *m*, H-9), 1.04 (3H, *d*, 6.5, H-21), 0.95 (3H, *s*, H-19). MS (*m*/*z*): 373.4399 [M + H]^+^; calculated for C_24_H_41_N_2_O^+^: 373.3219.

Kurchinin **18**: ^1^H-NMR (600 MHz CDCl_3_; δ (ppm), intensity, mult., *J* (Hz)): 7.74 (1H, *d*, 10.3, H-1), 6.15 (1H, *dd*, 2.0, 10.3, H-2), 6.10 (1H, *t*, 1.6, H-4), 4.10 (1H, *tt*, 6.1, 10.1, H-11), 2.52 (1H, *dd*, 5.0, 1.3, H-16α), 2.49 (1H, *dt*, 3.6, 1.3, H-6β), 2.47 (1H, *dt*, 7.7, 1.2, H-16β), 2.43 (1H, *dq*, 4.3, 2.5, H-6α), 2.22 (1H, *dt*, 5.0, 12.5, H-12α), 2.08 (1H, *ddd*, 6.4, 4.0, 1.5, H-7α), 1.96 (1H, *dddd*, 12.5, 9.0, 5.9, 1.2, H-15α), 1.83 (1H, *dtd*, 11.0, 3.8, 12.1, H-8), 1.57 (1H, *m*, H-15β), 1.35 (1H, *ddd*, 11.7, 5.9, 1.3, H-14), 1.32 (3H, *s*, H-19), 1.25 (1H, *m*, H-12β), 1.17 (1H, *m*, H-7β), 1.16 (1H, *m*, H-9), 0.95 (3H, *s*, H-18). MS (*m*/*z*): 301.3773 [M + H]^+^; calculated for C_19_H_25_O_3_: 301.1804.

*N*^3^-Isopentenyl adenine **19**: ^1^H-NMR (600 MHz CDCl_3_; δ (ppm), intensity, mult., *J* (Hz)): 8.06 (1H, *s*, H-8), 8.01 (1H, *s*, H-2), 5.51 (1H, *m*, H-12), 5.00 (2H, *d*, 7.5, H-11), 1.85 (3H, *s*, H-14), 1.82 (3H, *s*, H-15). MS (*m*/*z*): 204.1475 [M + H]^+^; calculated for C_10_H_14_N_5_^+^: 204.1249.

All the spectra data were in full agreement with reported literature data [21,22,23,24,25,26,27,28,29,30,31].

### 3.7. In Vitro Biological Assays

In vitro tests for biological activity of the crude extracts, fractions, sub-fractions and isolated compounds against *Trypanosoma brucei rhodesiense* (bloodstream trypomastigotes, STIB 900 strain), *Trypanosoma cruzi* (amastigotes, Tulahuen C4 strain), *Leishmania donovani* (amastigotes, MHOM-ET-67/L82 strain), *Plasmodium falciparum* (intraerythrocytic form, NF54 strain) and cytotoxicity test against mammalian L6 cell line from rat skeletal myoblasts were performed according to the established standard protocol [33]. However, only the activity data against *Trypanosoma brucei rhodesiense* is reported due to the inactivity of the tested substances against other parasites at the concentration tested.

## 4. Conclusions

Steroid alkaloids from *Holarrhena africana* have proved to be an interesting new class of highly active and selective lead compounds against *Trypanosoma brucei rhodesiense.* Of the 17 steroid alkaloids isolated, eight were identified to possess significant antitrypanosomal activity with *IC*_50_ values well below 1 µM and represent potential candidates for further study. Some SARs were observed which indicates that a ∆^5,6^ steroid nucleus with a monomethyl amino group at C-3 and a pyrrolidine ring with the amino nitrogen placed between C-20 and C-18 represents an optimum, among the compounds isolated so far, for high antitrypanosomal activity and selectivity. This essential pharmacophoric requirement for active and selective anti-trypanosomal activity is currently under refinement in terms of 3D QSAR modelling; this modeling will provide more detailed explanations to the present findings and possibly allow activity predictions for yet untested compounds of this type, aiming at structural optimization.

## Figures and Tables

**Table 1:** In vitro anti-trypanosomal and cytotoxic activity of crude extracts, alkaloid fraction and CC fractions of *H. africana* leaves and stem bark.

Tested Substance	Leaves			Stem bark		
	*Tbr*	Cytotox. L6	SI	*Tbr*	Cytotox. L6	SI
Crude extract	4.70 ^c^	87.5 ^c^	18.6	0.708 ± 0.013	>100	>100
Alk. fraction	0.905 ± 0.405	43.8 ^c^	48.4	0.143 ± 0.091	16.85 ± 1.344	117.8
Polar fraction	54.4 ^c^	n.t.	n.a.	35.2 ± 25.4	>100	n.a.
Fraction 11	1.87 ± 1.8	n.t.	n.a.	0.960 ± 0.325 ^a^	>100	>100
Fraction 12	0.191 ± 0.001	n.t.	n.a.	0.225 ± 0.012 ^b^	>100	>100
Fraction 13	0.031 ± 0.005	n.t.	n.a.	n.a.	n.a.	n.a.
Fraction 14	0.219 ± 0.004	n.t.	n.a.	n.a.	n.a.	n.a.
Pos. control	0.002 ± 0.001	0.004 ± 0.001	n.a.	0.002 ± 0.001	0.004 ± 0.001	n.a.

Data are average of two independent determinations, *IC*_50_ ± deviation from mean value, n.a. = not applicable, n.t. = not tested, ^a^ fraction HF8, ^b^ fraction HF9, ^c^ one replicate was used to determine *IC*_50_, positive controls: melarsoprol (*Tbr*), podophyllotoxin (cytotoxic L6), selectivity indices (SI) = cytotoxic *IC*_50_ (L6)/*IC*_50_*Tbr*.

**Table 2:** In vitro anti-trypanosomal activity and cytotoxicity of isolated alkaloids.

Compounds		*Tbr* (STIB900)		Cytotoxicity L6 Cells		SI
		*IC*_50_ (µg/mL)	*IC*_50_ (µM)	*IC*_50_ (µg/mL)	*IC*_50_ (µM)	
3β-Holaphyllamine	(**1**)	0.127 ± 0.088	0.402 ± 0.281	1.61 ± 0.21	5.10 ± 0.65	12.6
Holaphyllamine acetamid	(**2**)	1.73 ± 0.47	4.83 ± 1.33	5.49 ± 0.33	15.4 ± 0.9	3.2
*N*-methylholaphyllamine	(**3**)	0.025 ± 0.001	0.075 ± 0.004	0.829 ± 0.124	2.48 ± 0.44	33.2
3α-Holaphyllamine	(**4**)	0.117 ± 0.050	0.370 ± 0.159	5.00 ± 0.37	15.87 ± 1.17	42.9
3β-Dihydroholaphyllamine	(**5**)	0.213 ± 0.008	0.672 ± 0.027	5.51 ± 0.43	17.37 ± 1.36	25.8
3α-Dihydroholaphyllamine	(**6**)	0.382 ± 0.238	1.21 ± 0.75	5.38 ± 0.39	17.0 ± 1.2	14.1
Holadienine	(**7**)	4.81 ± 0.68	14.9 ± 2.1	n.t.	n.t.	n.t.
Holonamine	(**8**)	2.82 ± 0.76	8.67 ± 2.33	21.1 ± 7.5	64.9 ± 23.1	7.5
Cona-4,6-dienin-3-one	(**9**)	5.69 ± 0.07	17.5 ± 0.2	41.9 ± 19.9	129 ± 61	7.4
Cona-3,5-dienin-7-one	(**10**)	2.40 ± 0.30	7.37 ± 0.94	13.6 ± 6.2	41.7 ± 18.9	5.7
Conessimine	(**11**)	0.057 ± 0.028	0.167 ± 0.083	17.3 ± 4.0	50.4 ± 11.8	302
Isoconessimine	(**12**)	0.056 ± 0.038	0.166 ± 0.112	9.38 ^a^	27.4	168
Conessine	(**13**)	0.149 ± 0.031	0.419 ± 0.087	21.8 ^a^	61.2	146
Holarrhesine	(**14**)	0.054 ± 0.038	0.119 ± 0.084	6.51 ^a^	14.3	121
Holarrhetine	(**15**)	0.777 ± 0.182	1.66 ± 0.39	45.8 ^a^	97.7	58.9
Holarrheline	(**16**)	2.94 ± 0.37	8.21 ± 1.03	64.8 ± 14.2	181 ± 40	22.0
Holarrhenine	(**17**)	7.78 ± 2.23	20.9 ± 6.0	46.4 ± 12.8	125 ± 34	6.0
kurchinin	(**18**)	3.64 ± 0.21	12.1 ± 0.7	n.t.	n.t.	n.a.
Isopentenyl adenine	(**19**)	5.03 ± 0.54	24.78 ± 0.84	61.30 ± 4.24	302 ± 6.80	12.2

Data represent the average of two independent determinations, *IC*_50_± absolute deviation from mean value, n.a. = not applicable, n.t. = not tested, ^a^ one replicate was used to determine *IC*_50_, Positive controls are shown in Table 1.

## Supplementary Materials

Supplementary materials are available online. The ^1^H and ^13^C NMR of all the isolated compounds are provided as supplementary Figures S1–S38.
