# Supplementary material for: Steroid Alkaloids from Holarrhena africana with Strong Activity against Trypanosoma brucei rhodesiense
Source: Molecules. 2017 Jul 6;22(7):1129. doi: 10.3390/molecules22071129 (PMC6152089; doi:10.3390/molecules22071129)
Supplement: Supplementary file 1 [file molecules-22-01129-s001.pdf]

Supplementary Material

Steroid Alkaloids from *Holarrhena africana* with strong activity against *Trypanosoma brucei rhodesiense*

Charles O. Nnadi [1,2], Ngozi J. Nwodo [2], Marcel Kaiser [3,4], Reto Brun [3,4] and Thomas J. Schmidt [1,*]

[1] Institute of Pharmaceutical Biology and Phytochemistry (IPBP), University of Münster, PharmaCampus Corrensstraße 48, Münster D-48149, Germany; E-mail: charles.nnadi@unn.edu.ng (CON); thomschm@uni-muenster.de (TJS)

[2] Department of Pharmaceutical and Medicinal Chemistry, Faculty of Pharmaceutical Sciences, University of Nigeria Nsukka, 410001 Enugu State Nigeria; E-mail: ngozi.nwodo@unn.edu.ng (NJN)

[3] Swiss Tropical and Public Health institute (Swiss TPH), Socinstr. 57, Basel CH-4051, Switzerland; Email: marcel.kaiser@unibas.ch (MK); reto.brun@unibas.ch (RB)

[4] University of Basel, Petersplatz 1, Basel CH-4003, Switzerland

*Correspondence: thomschm@uni-muenster.de; Tel.: +49-251-83-33378

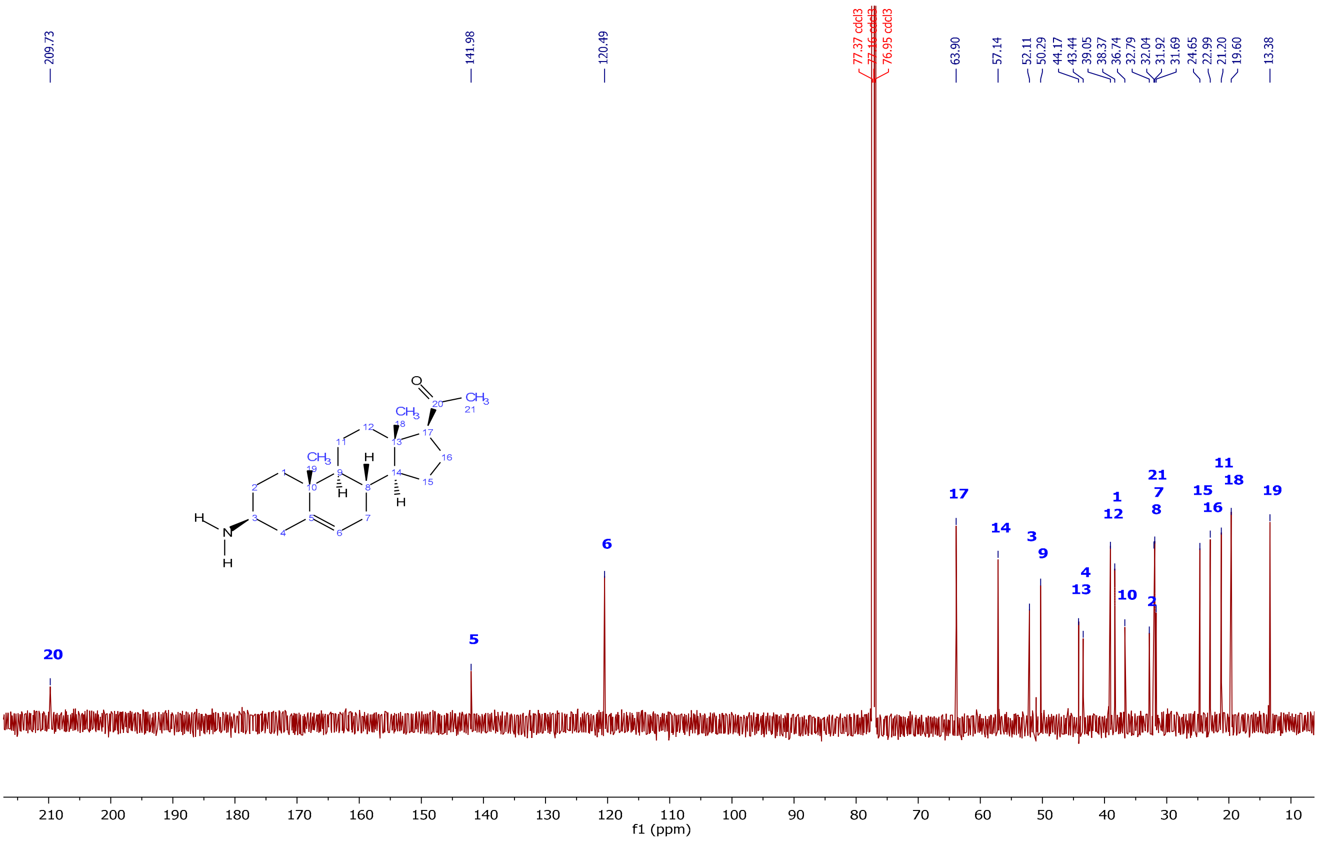

Figure S1: ^{13}C NMR spectrum of compound **1** ($CDCl_3$, 600 MHz)

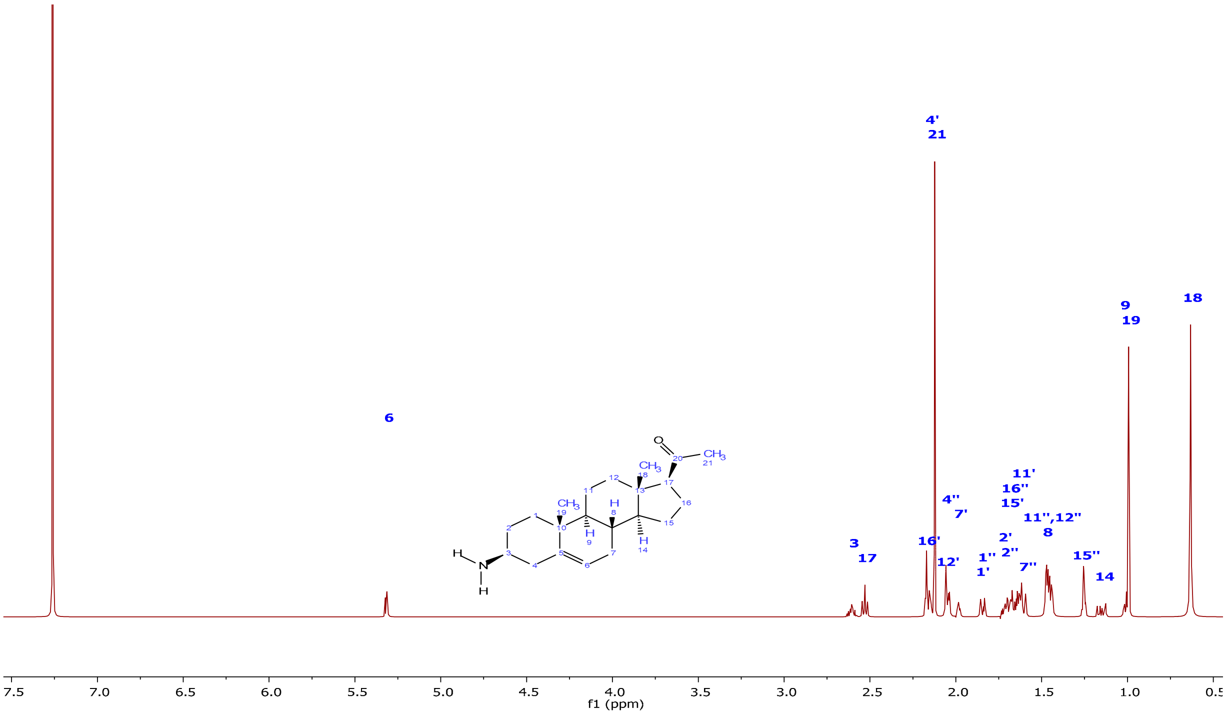

Figure S2: ^{1}H NMR spectrum of compound **1** ($CDCl_3$, 600 MHz)

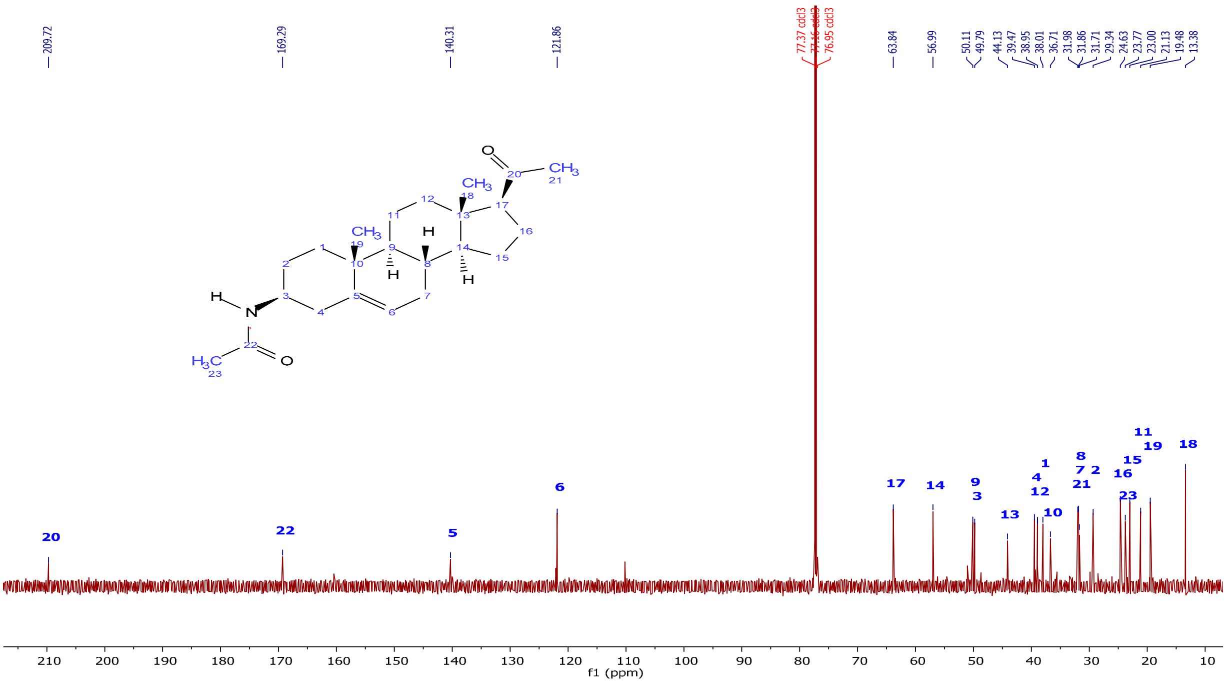

Figure S3: ^{13}C NMR spectrum of compound **2** ($CDCl_3$, 600 MHz)

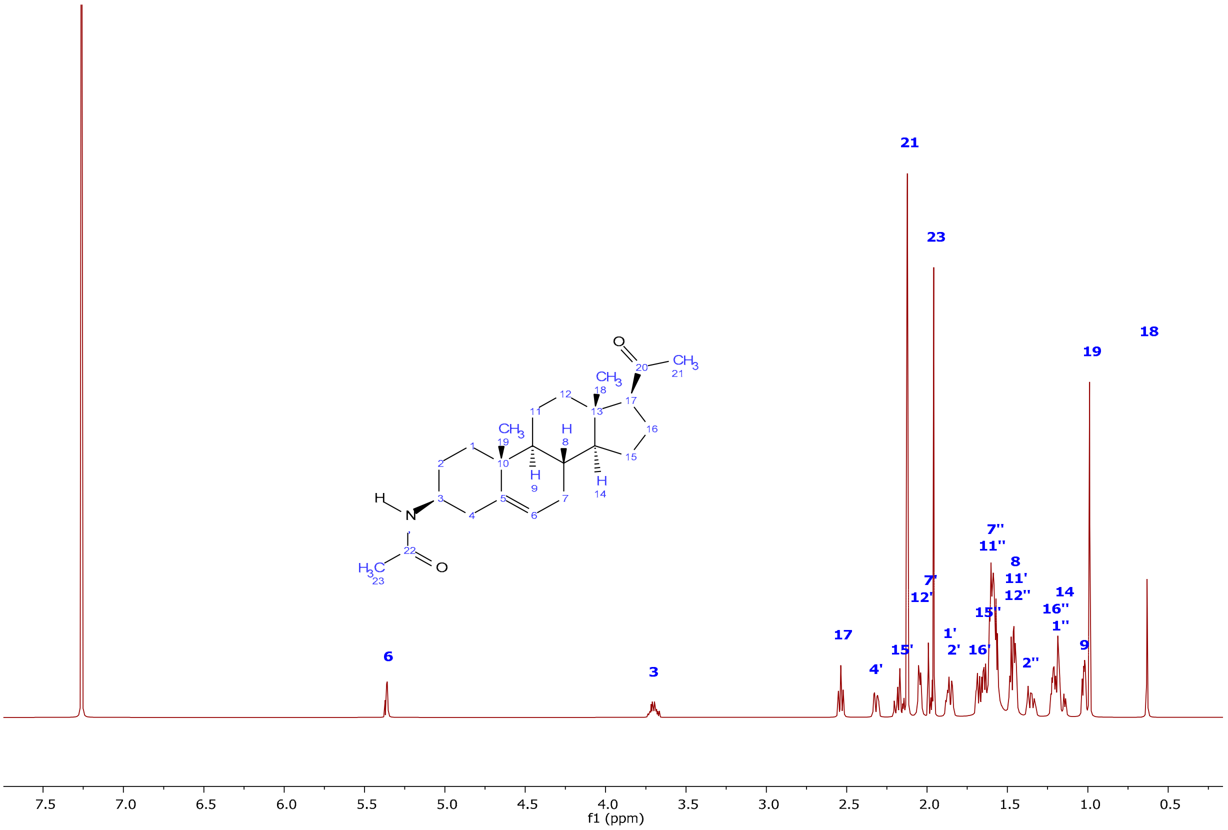

Figure S4: ^{1}H NMR spectrum of compound **2** ($CDCl_3$, 600 MHz)

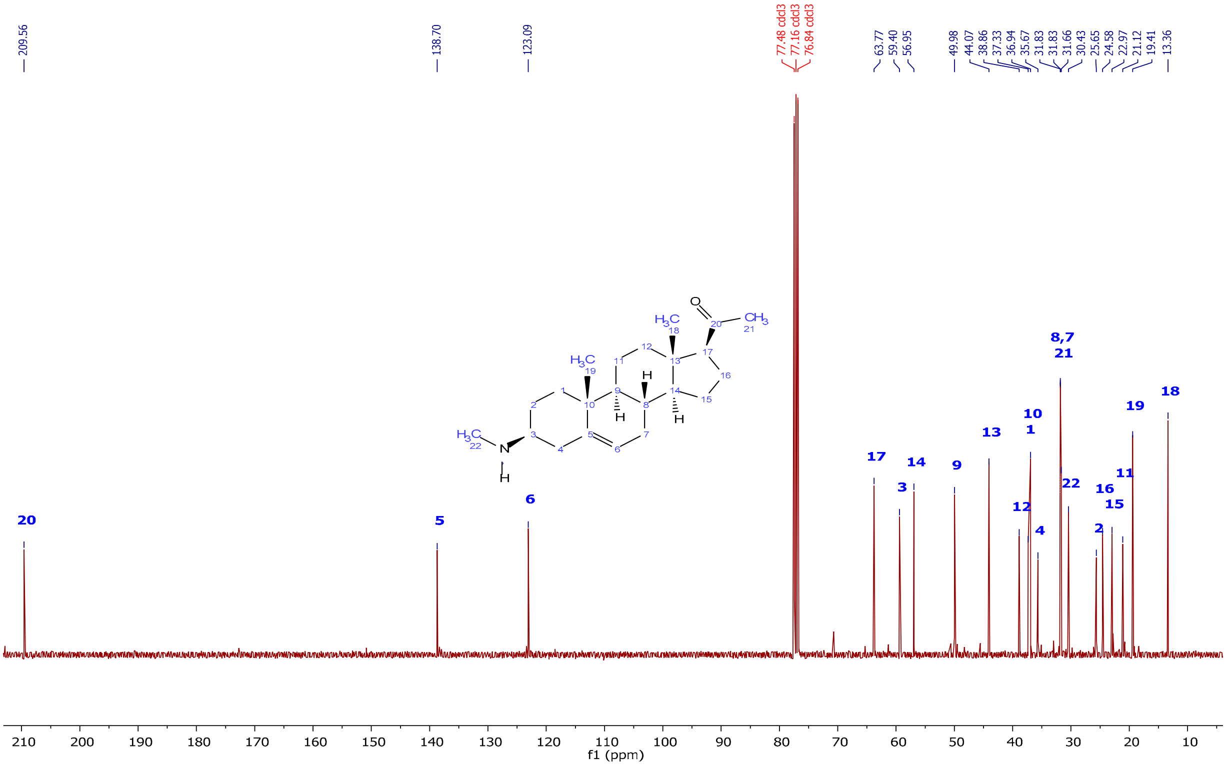

Figure S5: ^{13}C NMR spectrum of compound **3** ($CDCl_3$, 600 MHz)

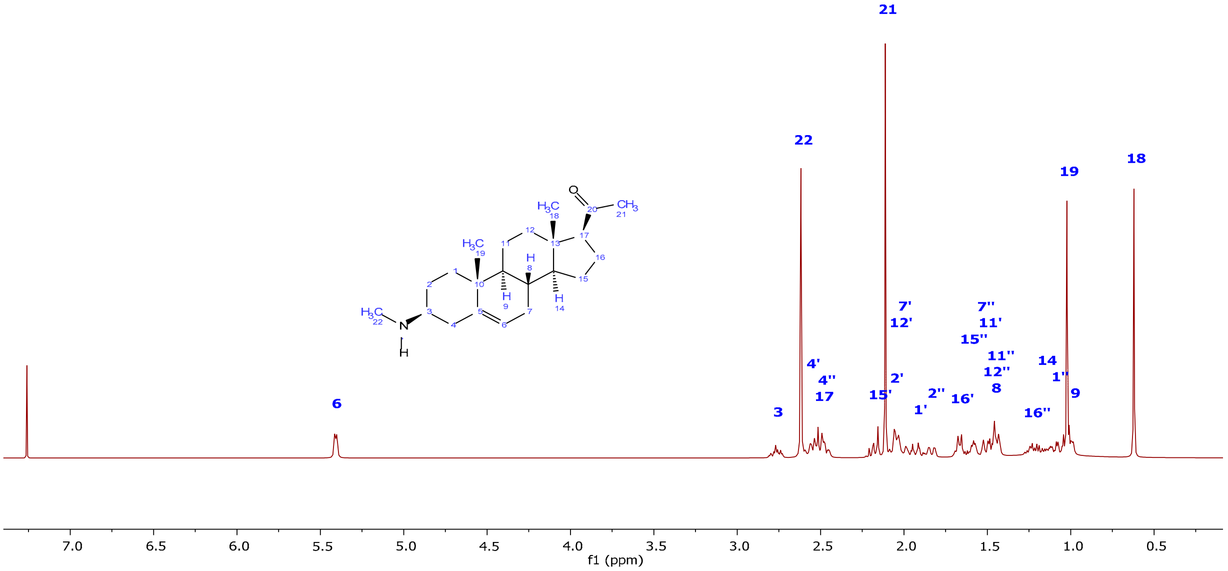

Figure S6: ^{1}H NMR spectrum of compound **3** ($CDCl_3$, 600 MHz)

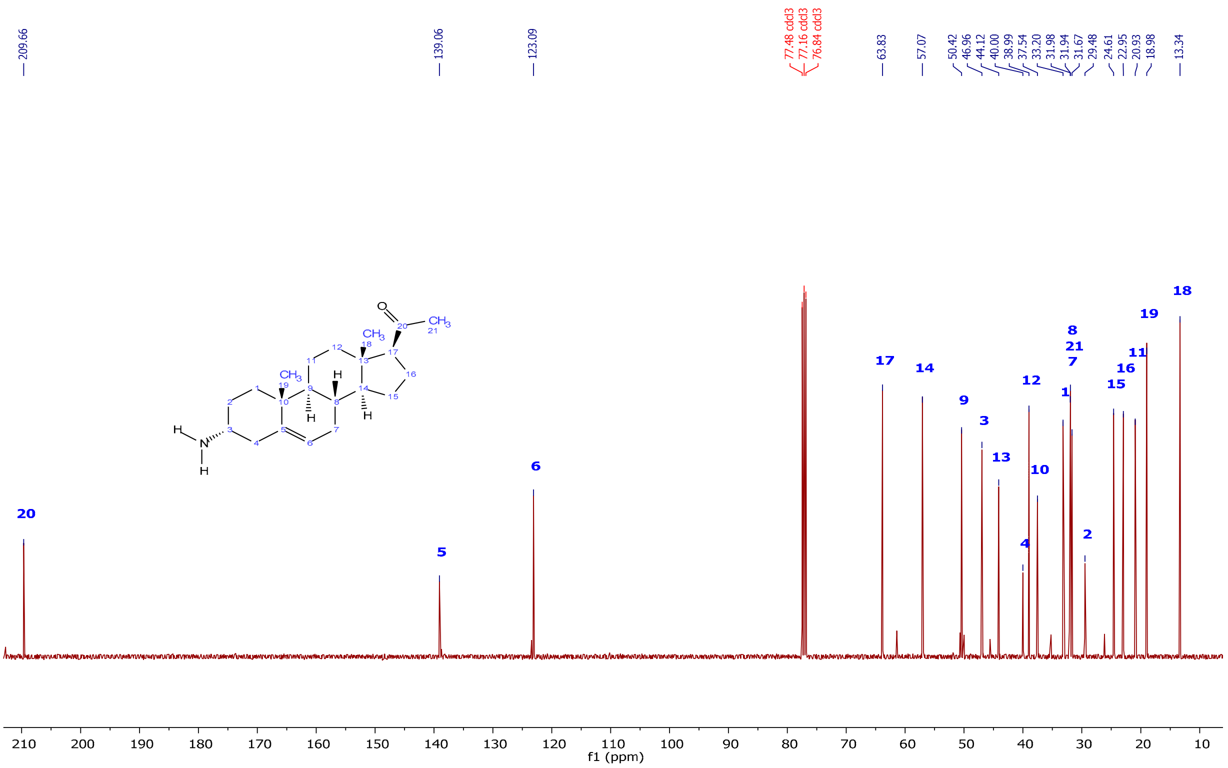

Figure S7: ^{13}C NMR spectrum of compound **4** ($CDCl_3$, 600 MHz)

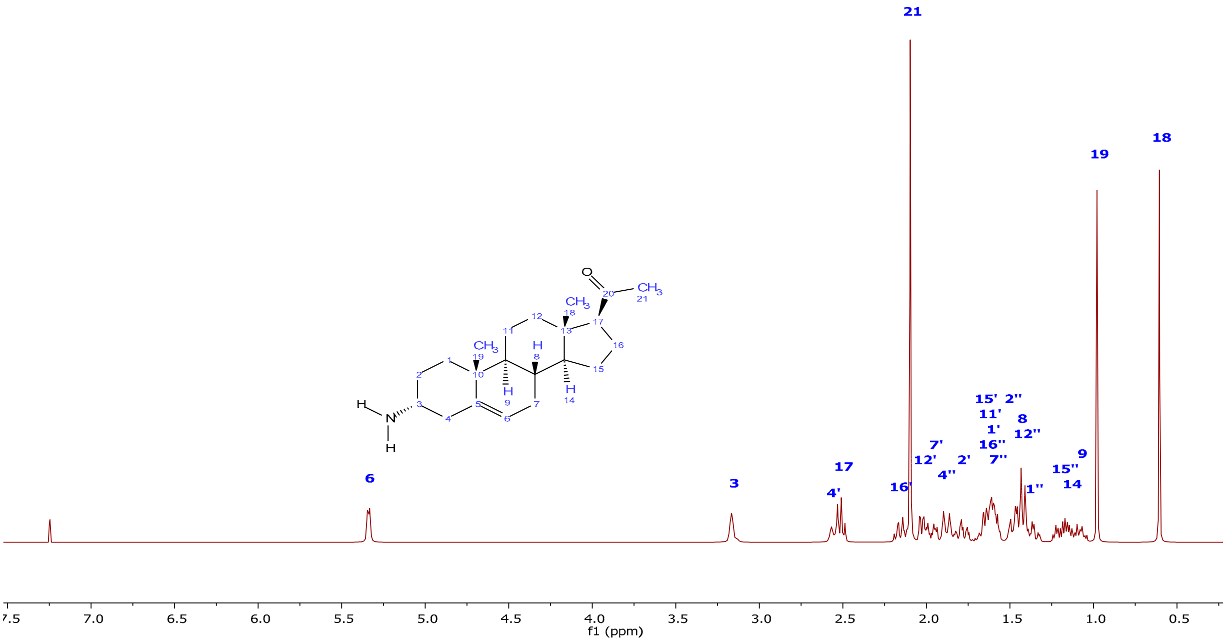

Figure S8: ^{1}H NMR spectrum of compound **4** ($CDCl_3$, 600 MHz)

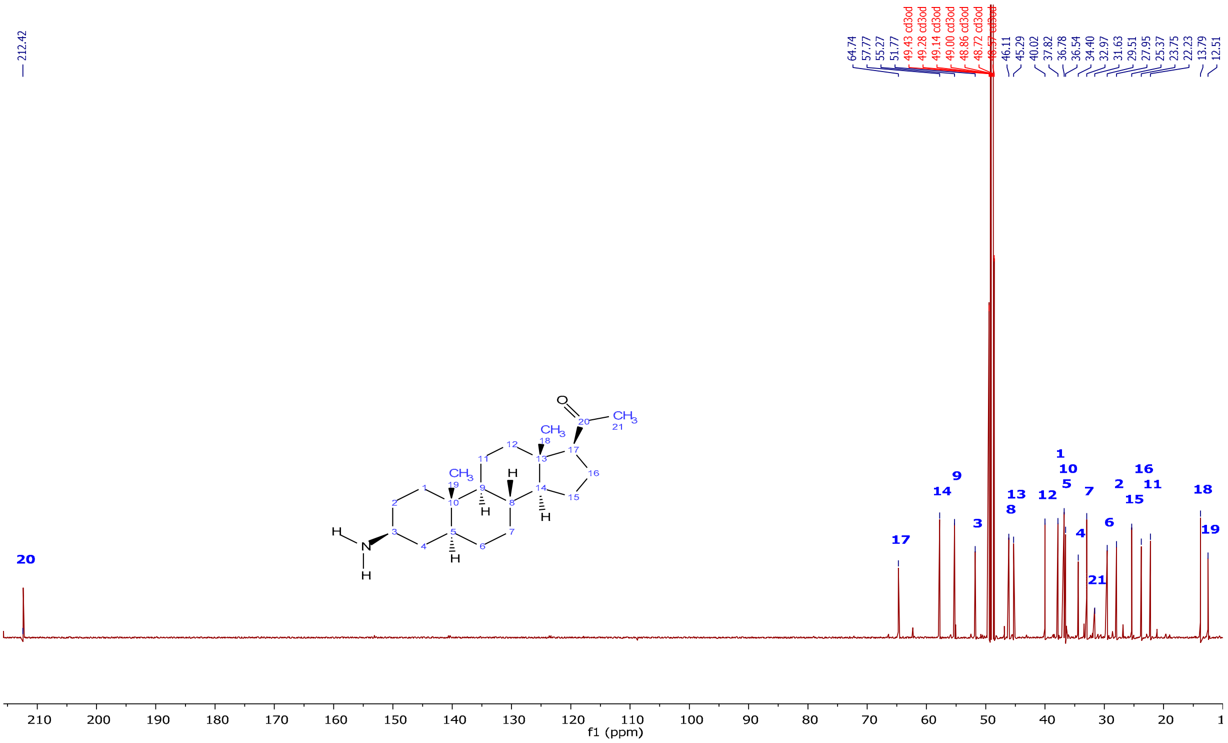

Figure S9: ^{13}C NMR spectrum of compound **5** (CD_3OD, 600 MHz)

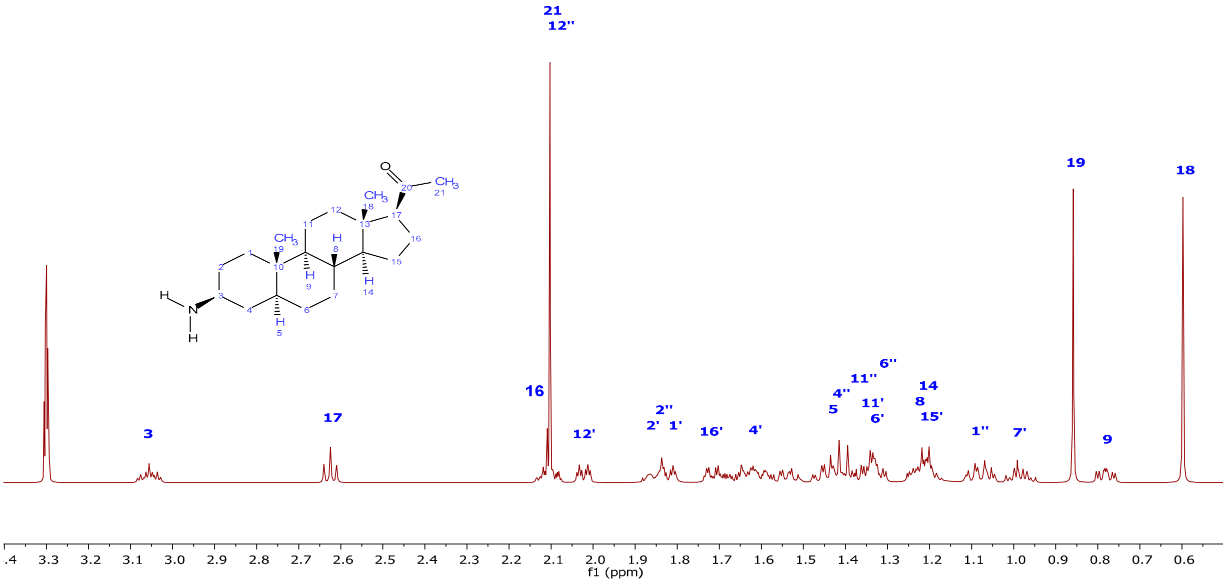

Figure S10: ^{1}H NMR spectrum of compound **5** (CD_3OD, 600 MHz)

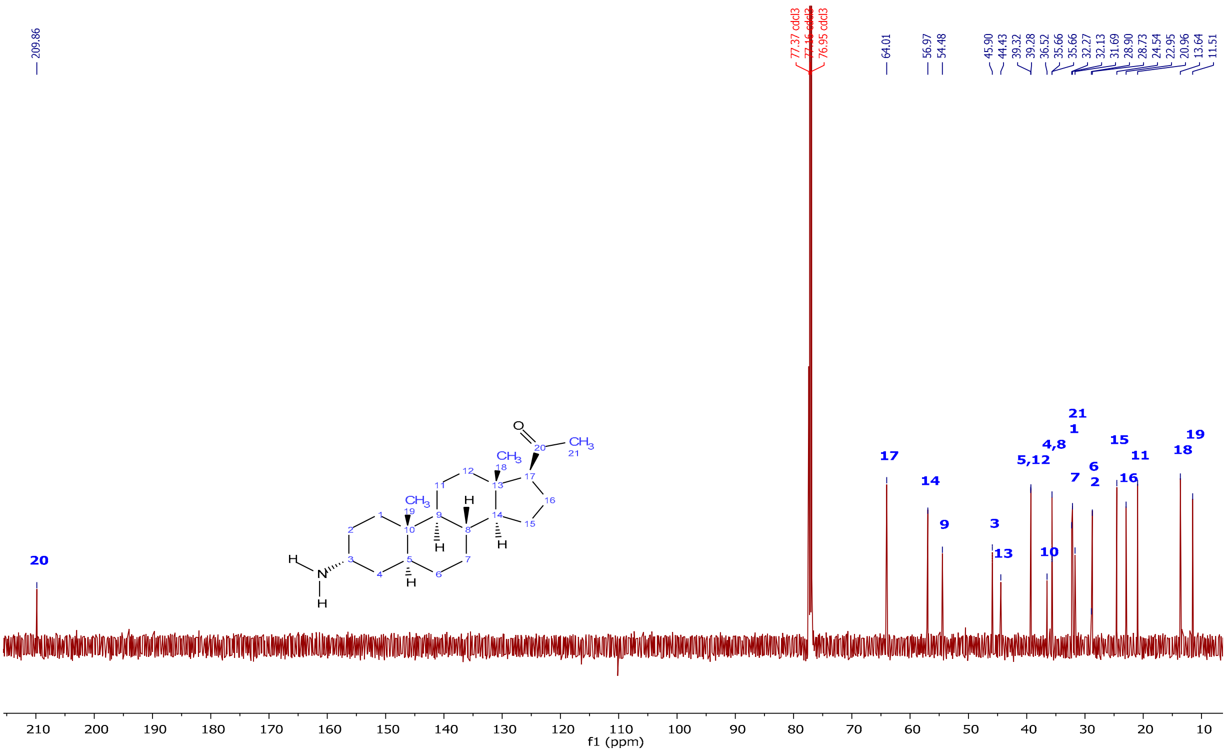

Figure S11: ^{13}C NMR spectrum of compound **6** ($CDCl_3$, 600 MHz)

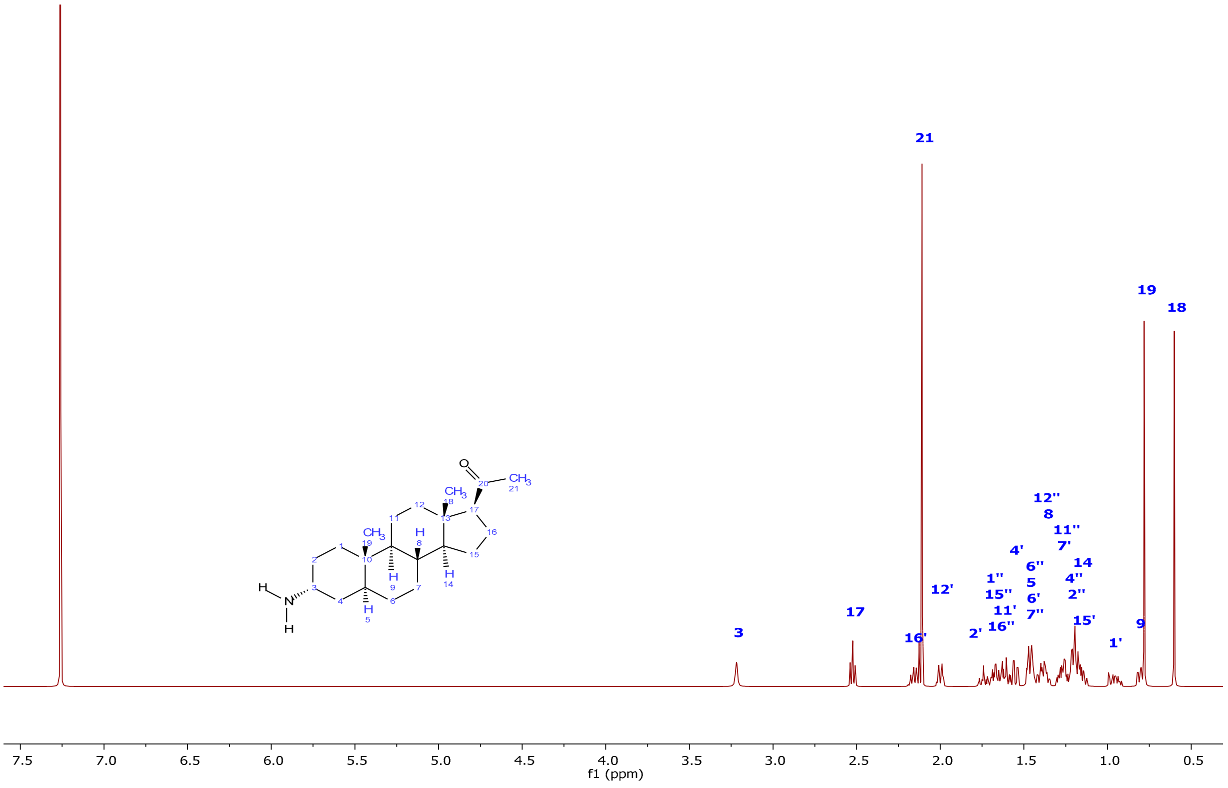

Figure S12: ^{1}H NMR spectrum of compound **6** ($CDCl_3$, 600 MHz)

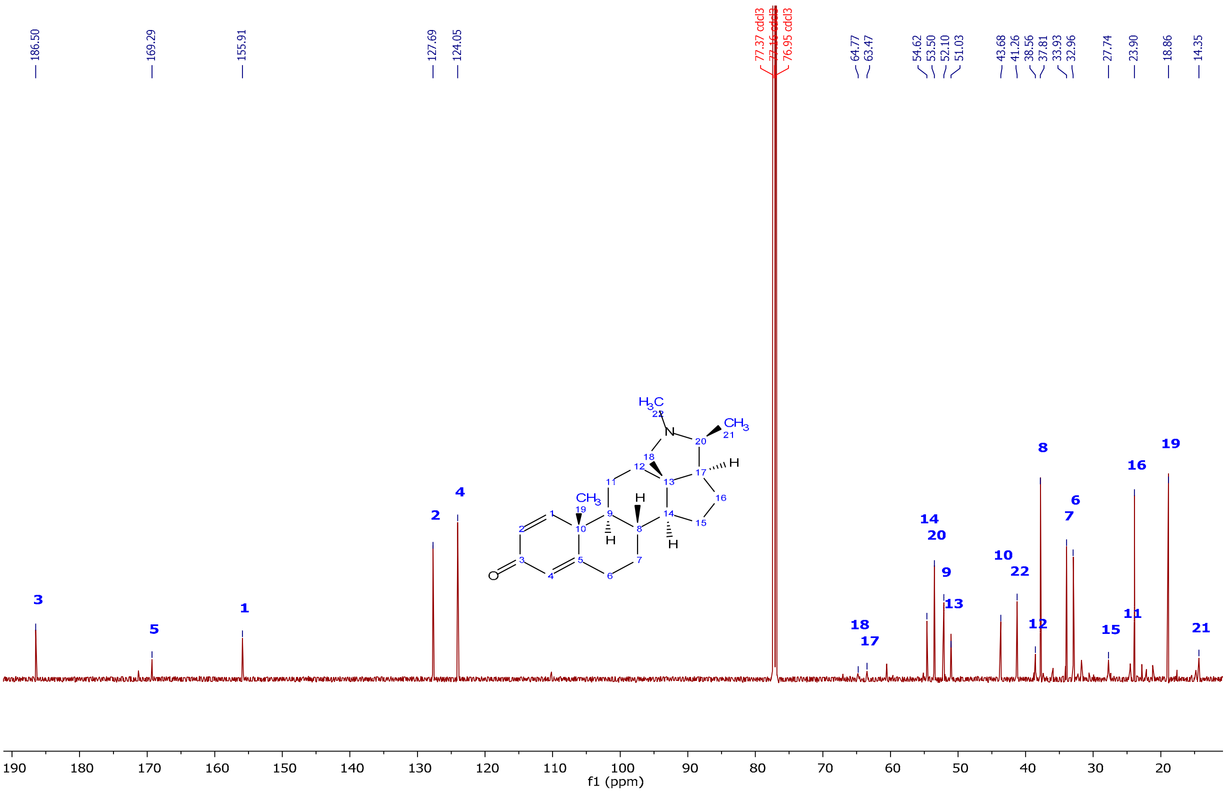

Figure S13: ^{13}C NMR spectrum of compound **7** ($CDCl_3$, 600 MHz)

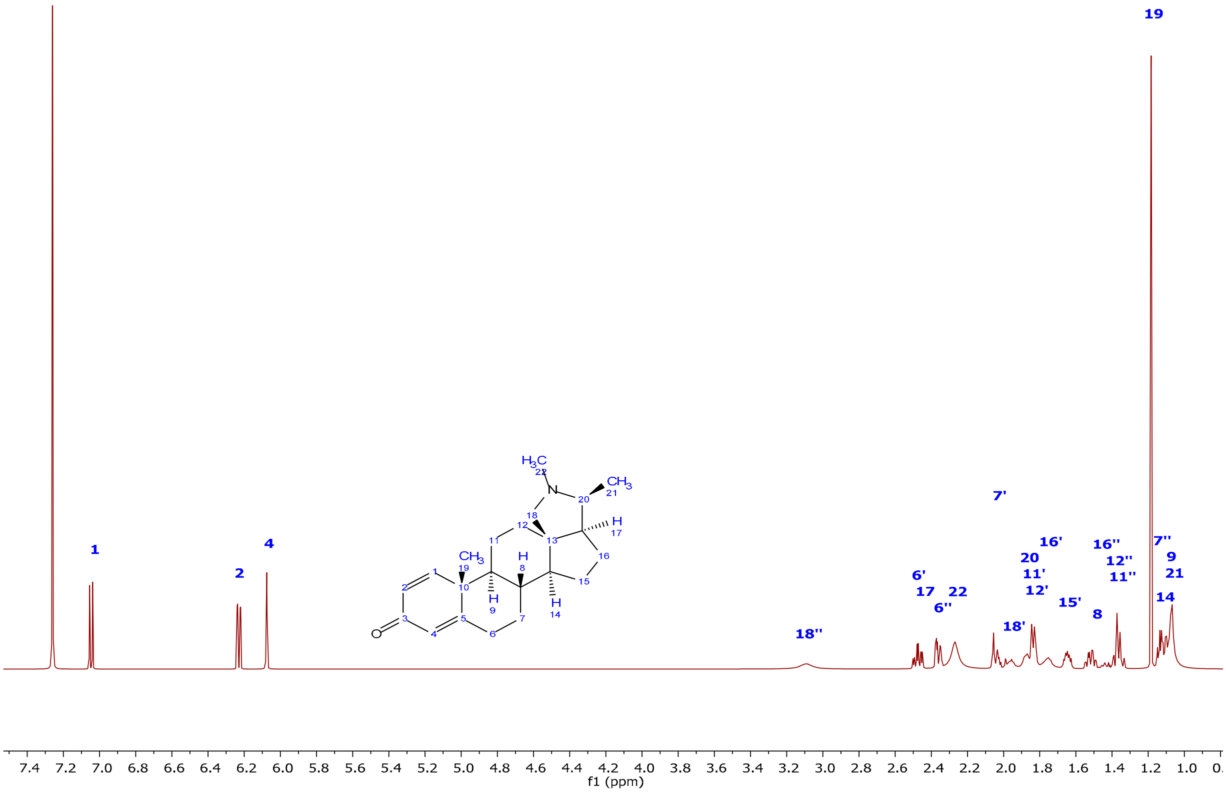

Figure S14: ^{1}H NMR spectrum of compound **7** ($CDCl_3$, 600 MHz)

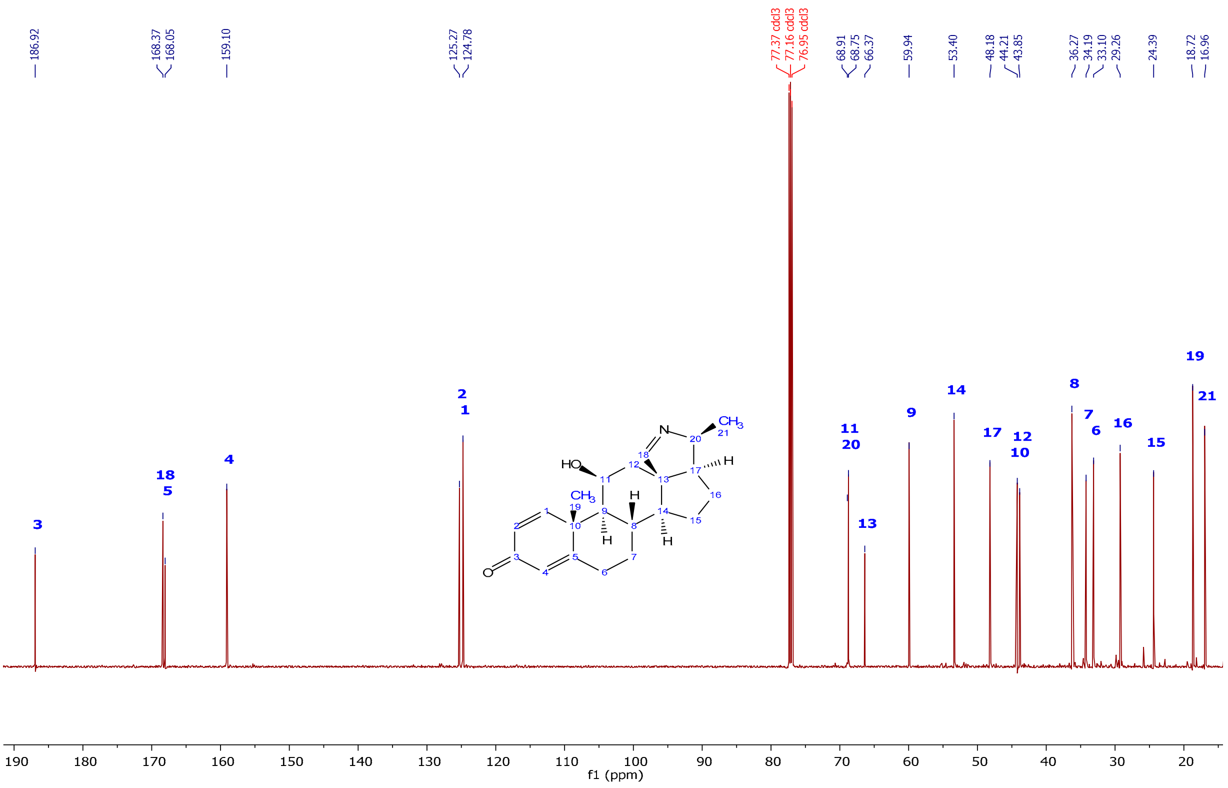

Figure S15: ^{13}C NMR spectrum of compound **8** ($CDCl_3$, 600 MHz)

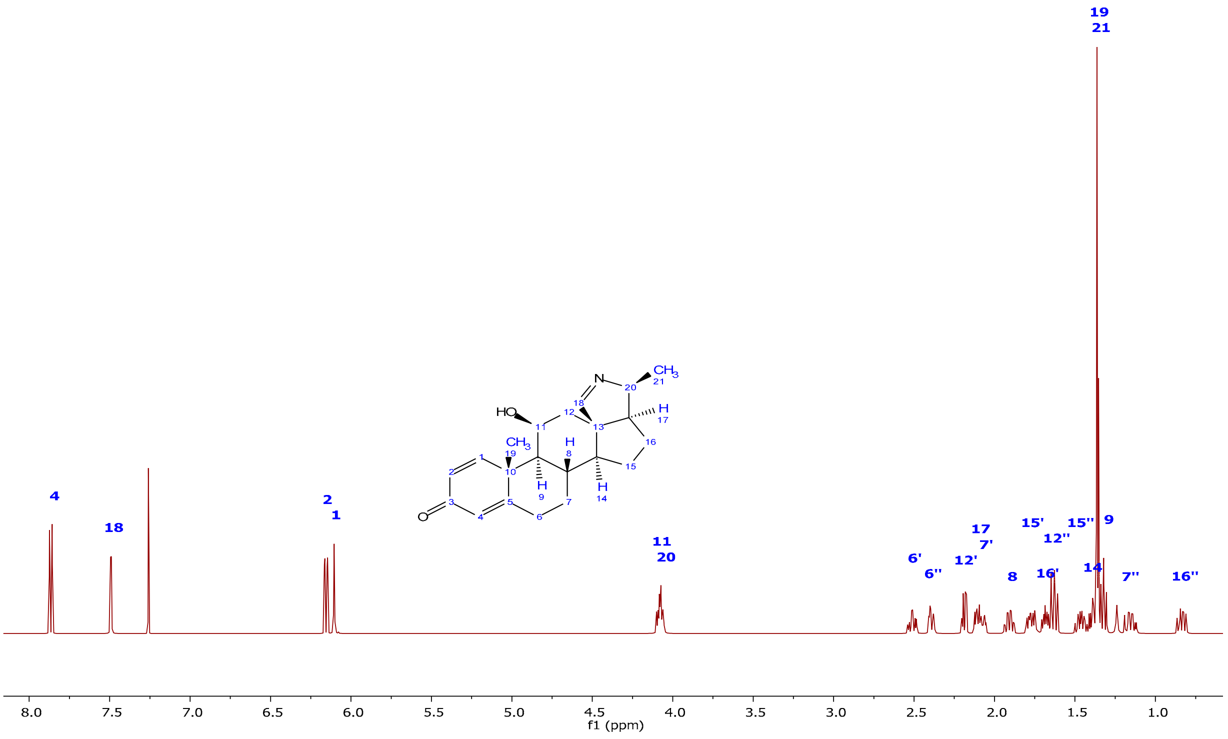

Figure S16: ^{1}H NMR spectrum of compound **8** ($CDCl_3$, 600 MHz)

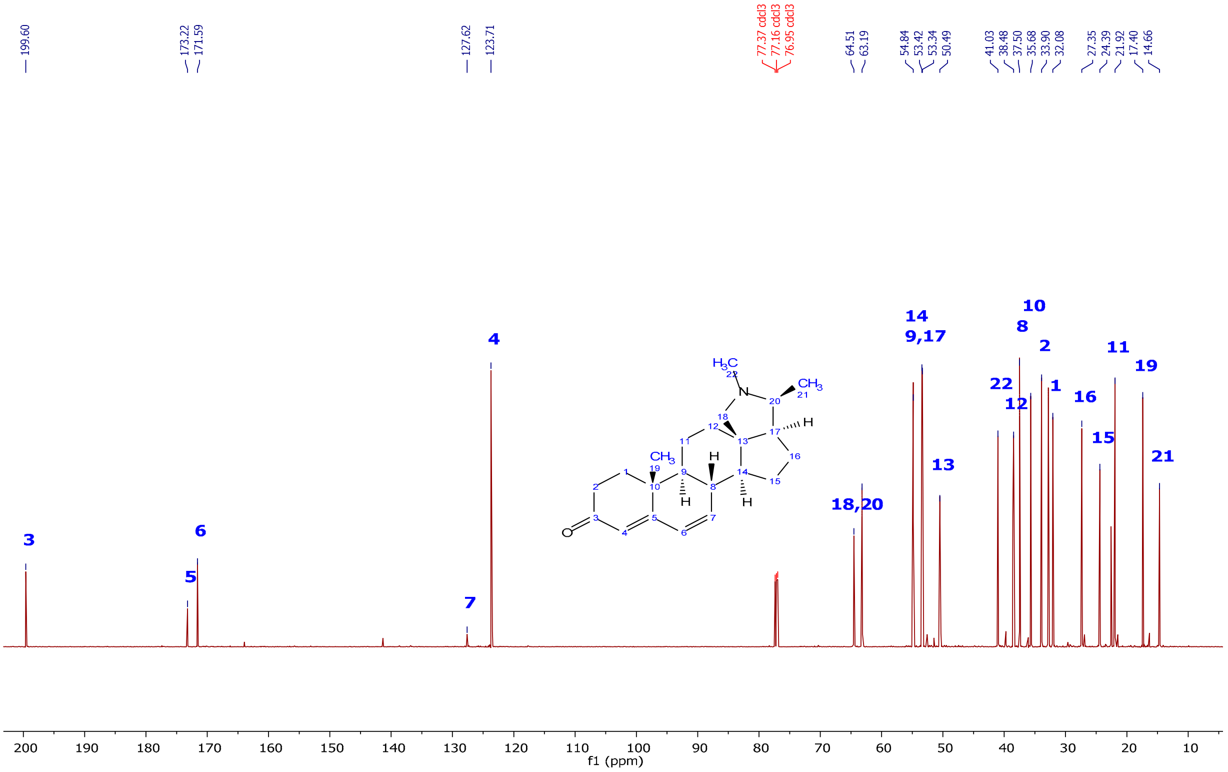

Figure S17: ^{13}C NMR spectrum of compound **9** ($CDCl_3$, 600 MHz)

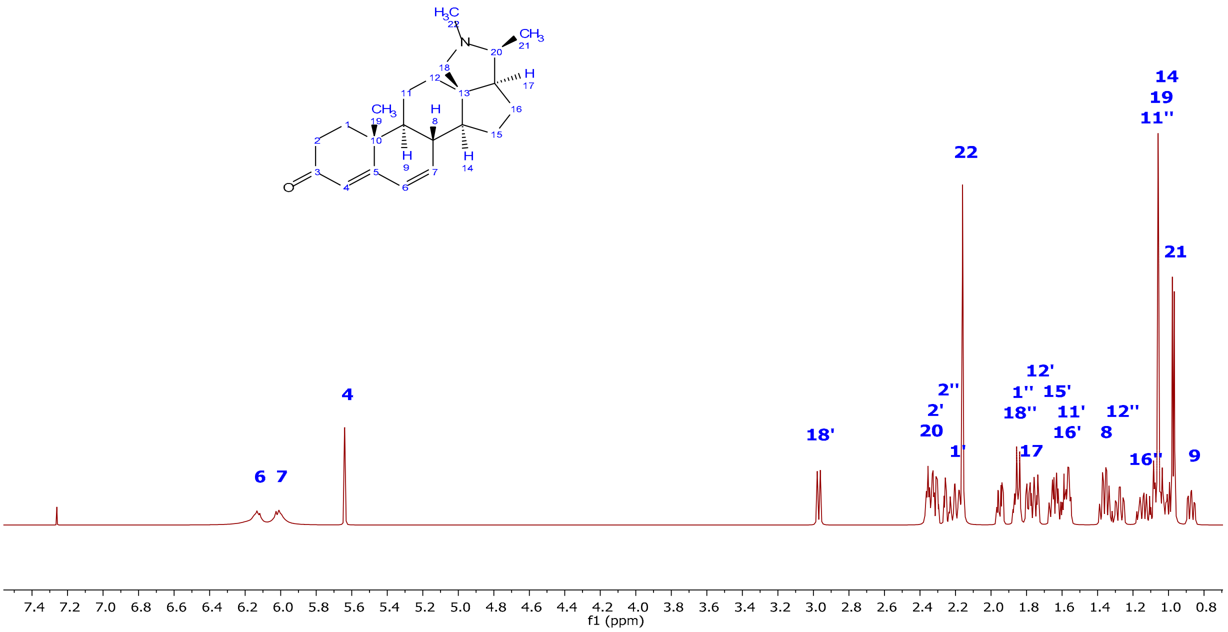

Figure S18: ^{1}H NMR spectrum of compound **9** ($CDCl_3$, 600 MHz)

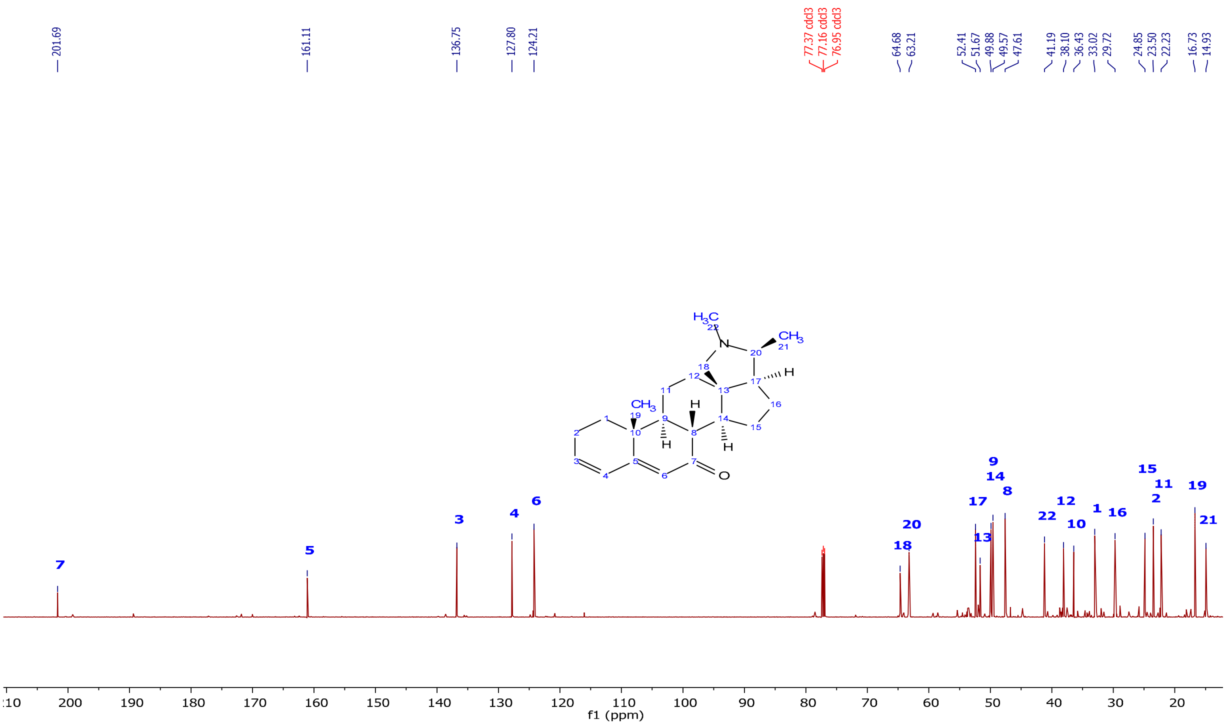

Figure S19: ^{13}C NMR spectrum of compound **10** ($CDCl_3$, 600 MHz)

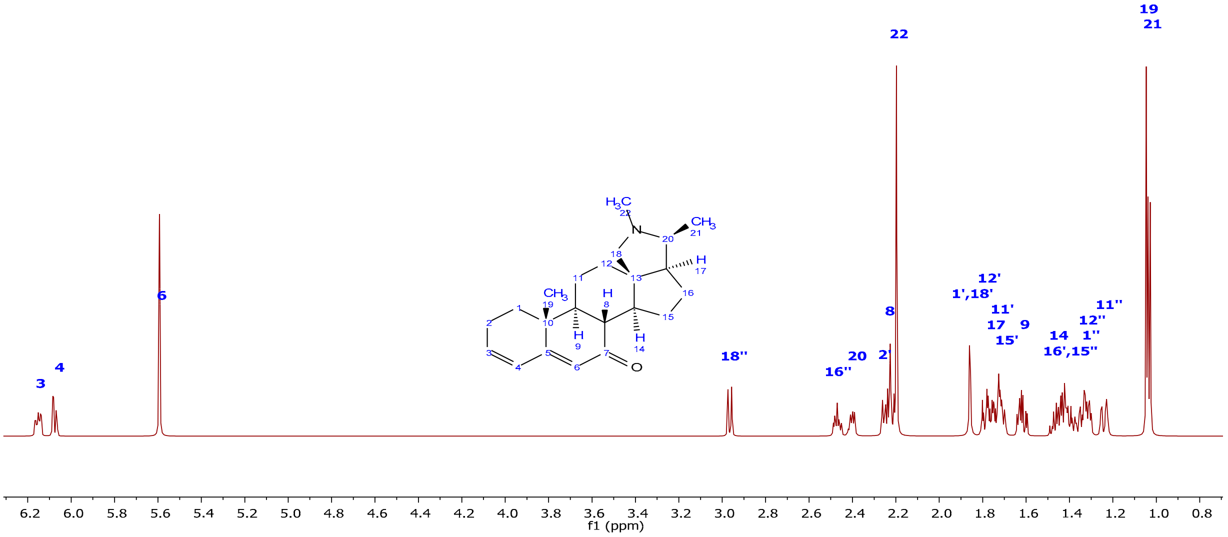

Figure S20: ^{1}H NMR spectrum of compound **10** ($CDCl_3$, 600 MHz)

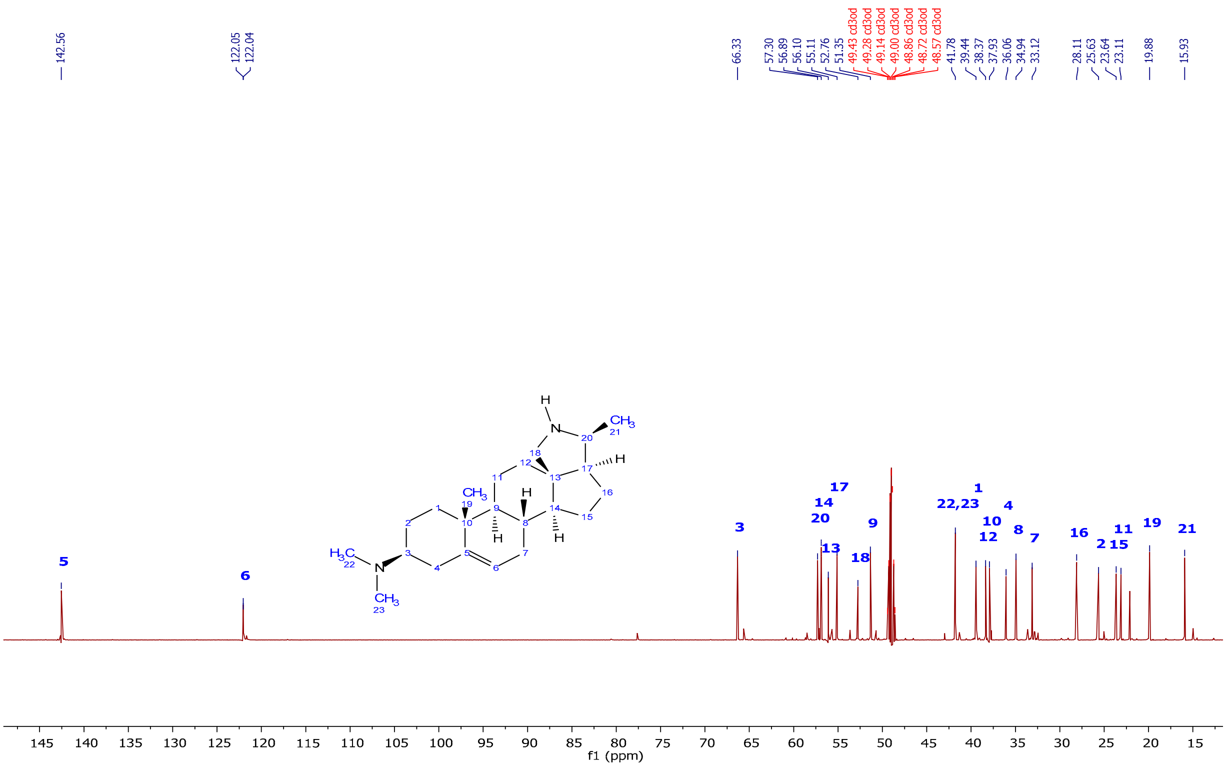

Figure S21: ^{13}C NMR spectrum of compound **11**(CD3OD, 600 MHz)

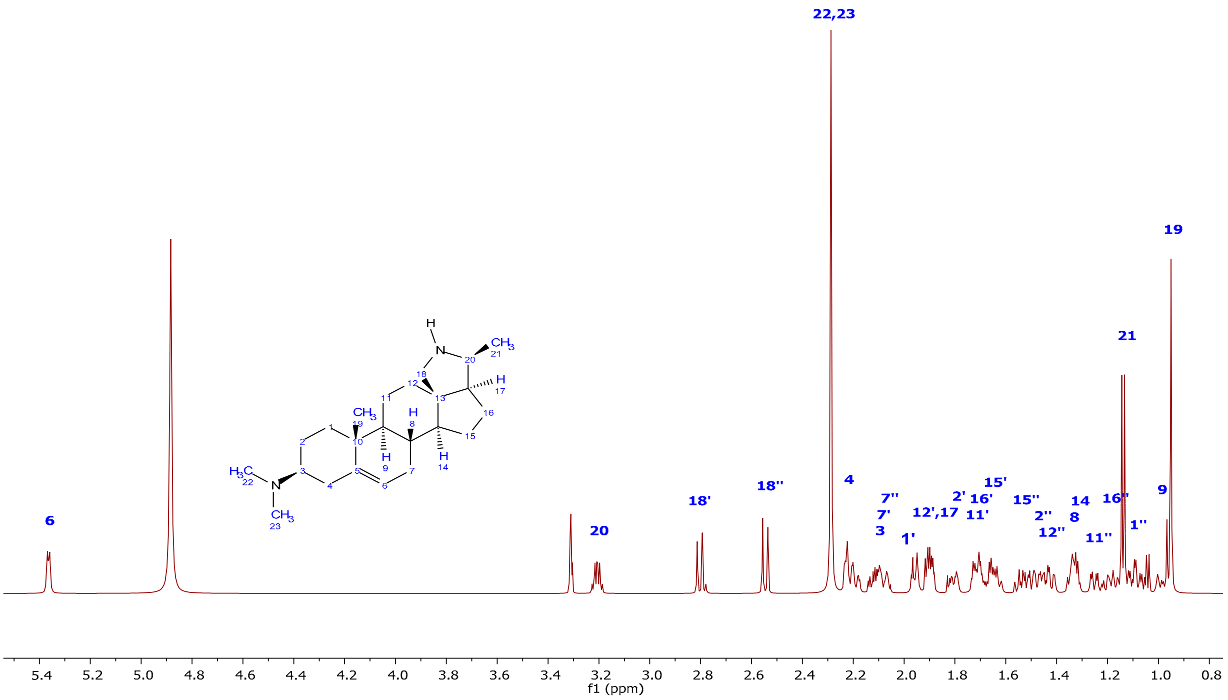

Figure S22: ^{1}H NMR spectrum of compound **11** (CDCl3, 600 MHz)

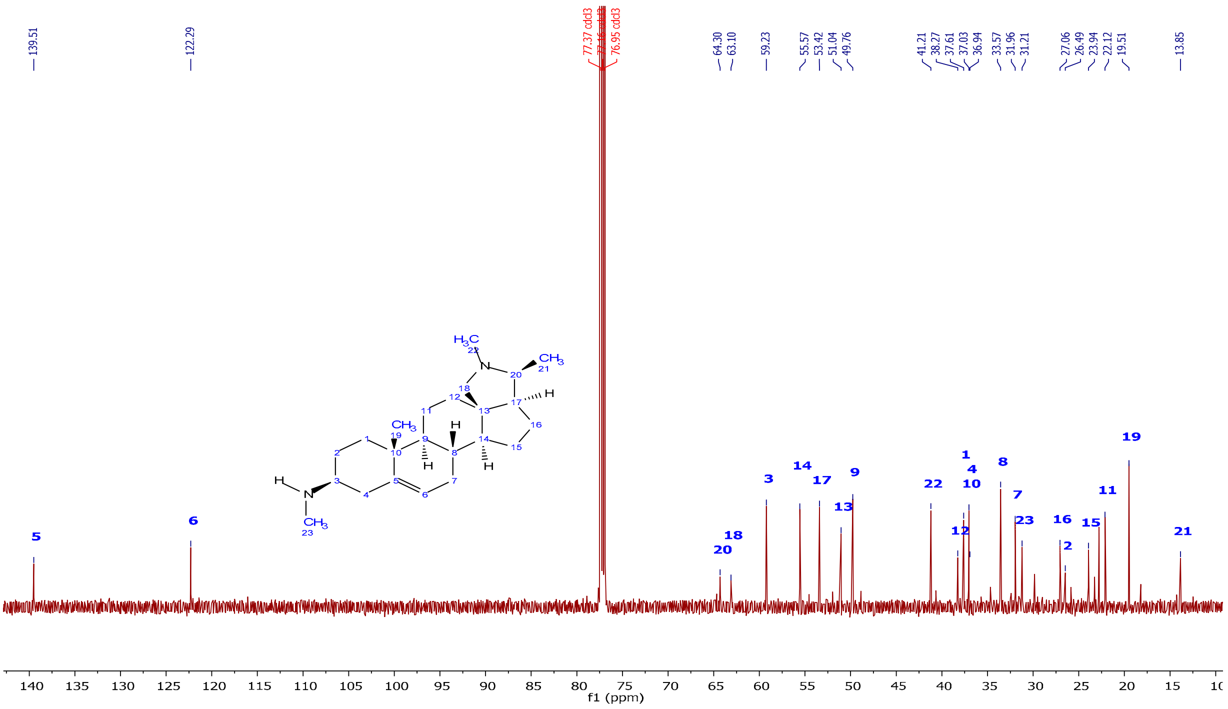

Figure S23: ^{13}C NMR spectrum of compound **12** ($CDCl_3$, 600 MHz)

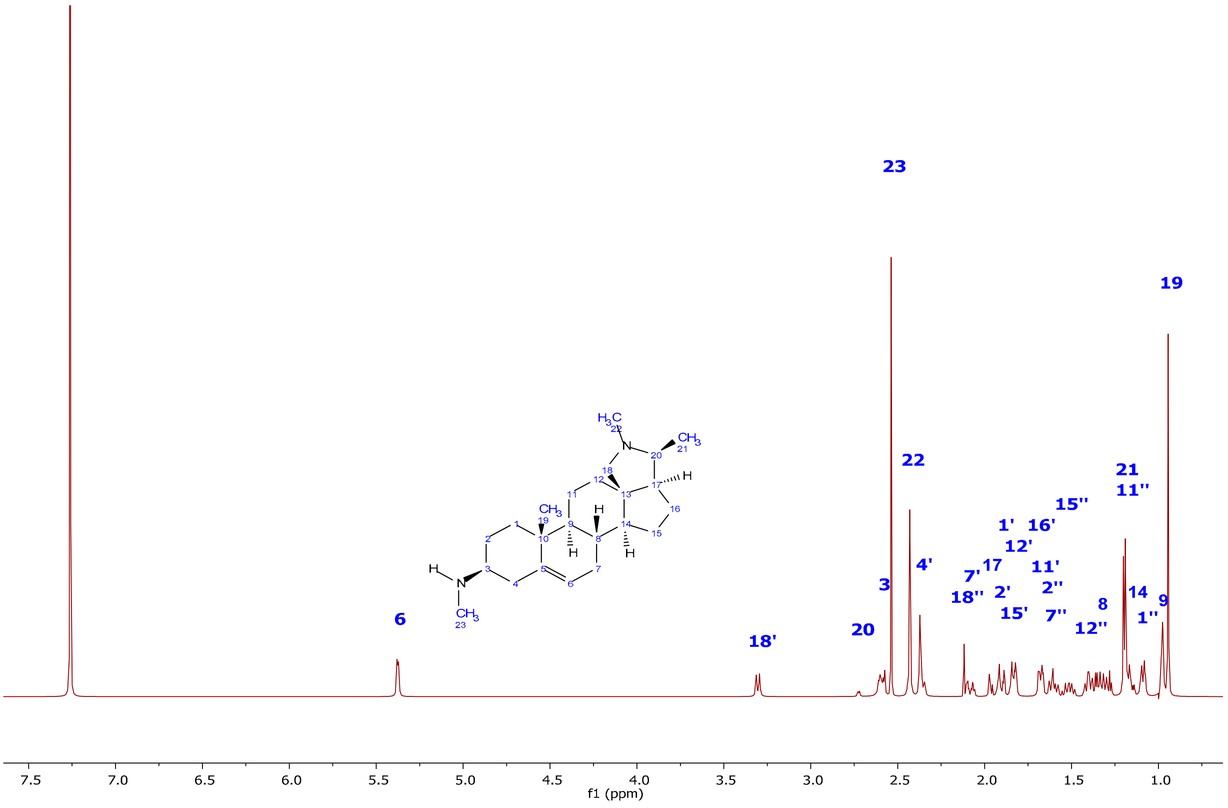

Figure S24: ^{1}H NMR spectrum of compound **12** ($CDCl_3$, 600 MHz)

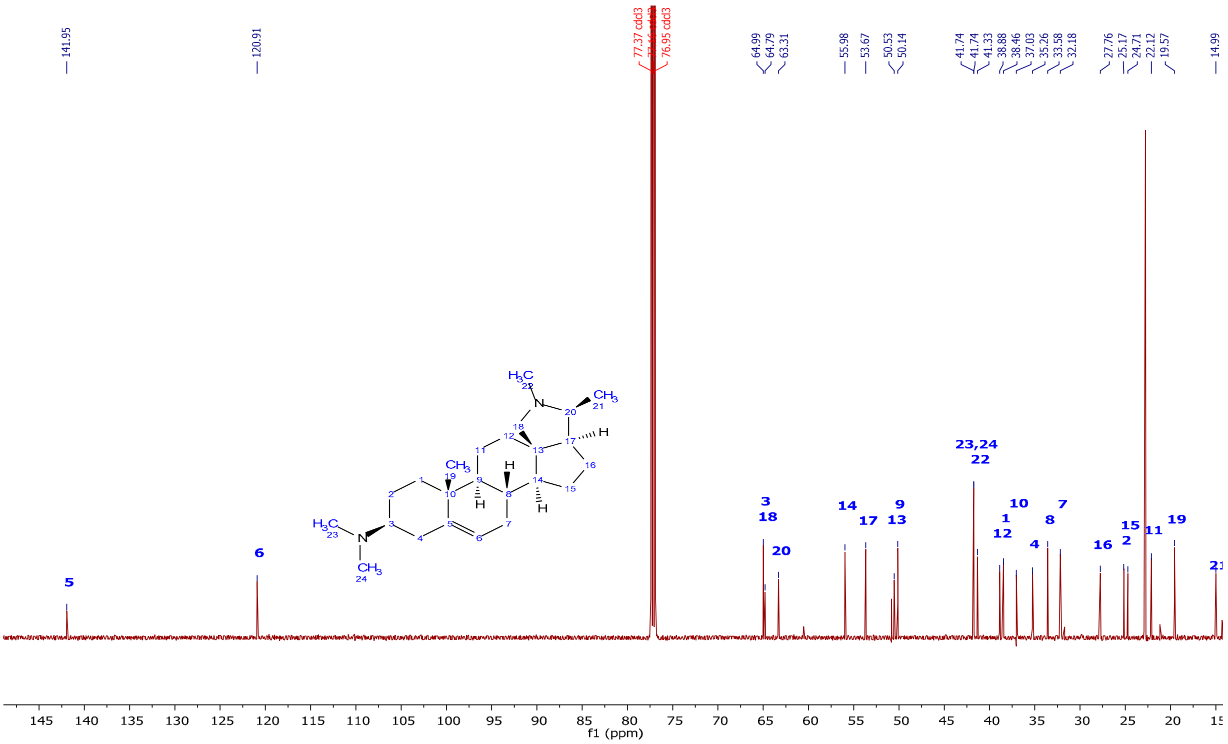

Figure S25: ^{13}C NMR spectrum of compound **13** ($CDCl_3$, 600 MHz)

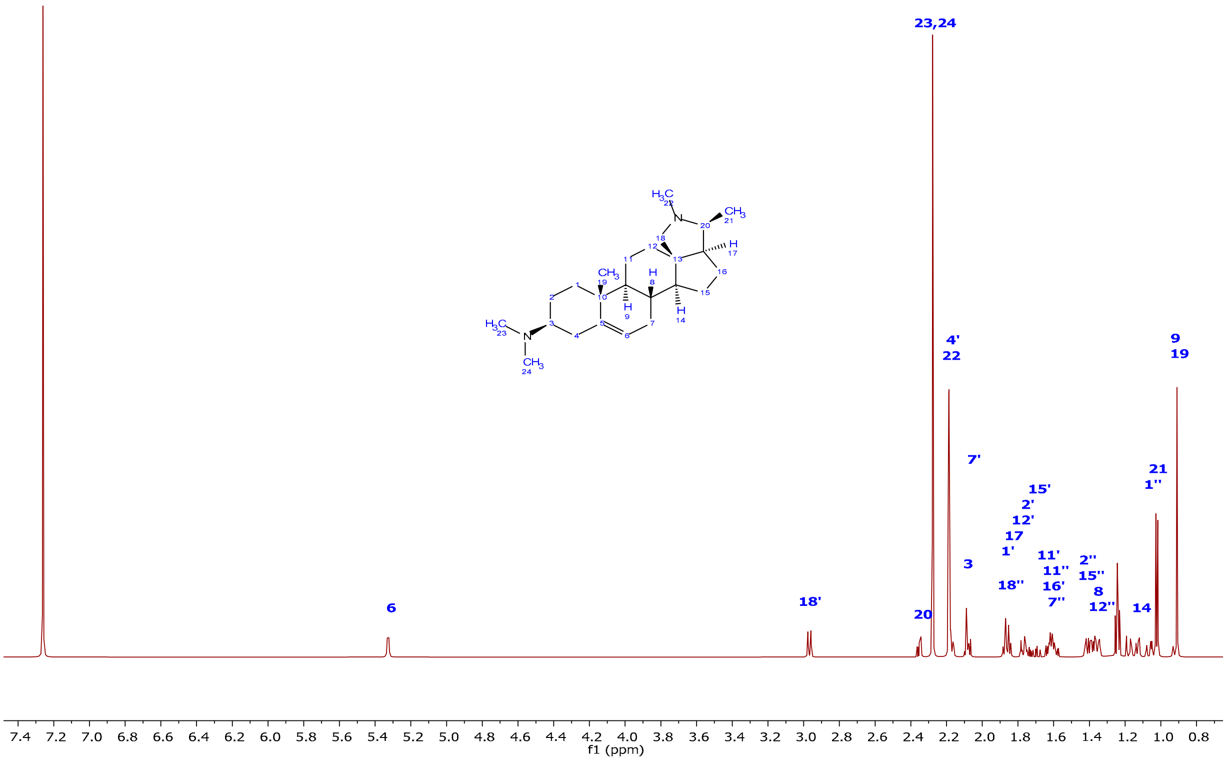

Figure S26: ^{1}H NMR spectrum of compound **13** ($CDCl_3$, 600 MHz)

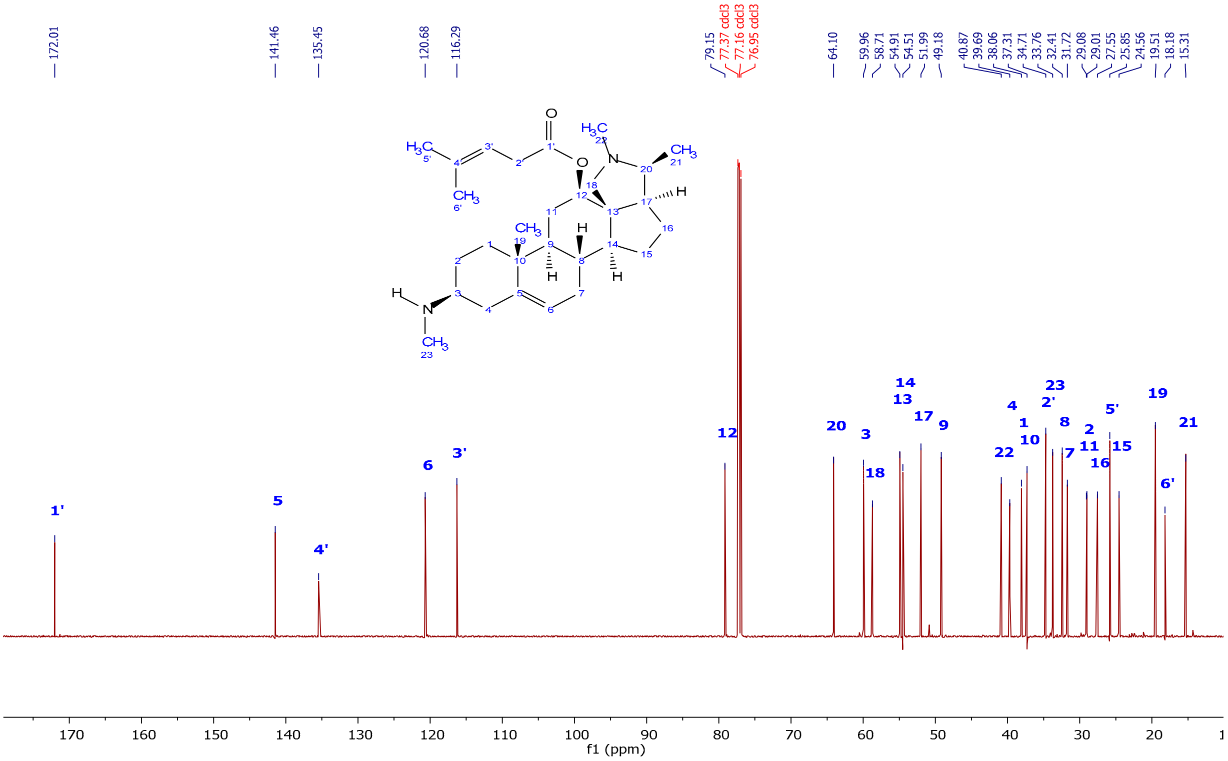

Figure S27: ^{13}C NMR spectrum of compound **14** ($CDCl_3$, 600 MHz)

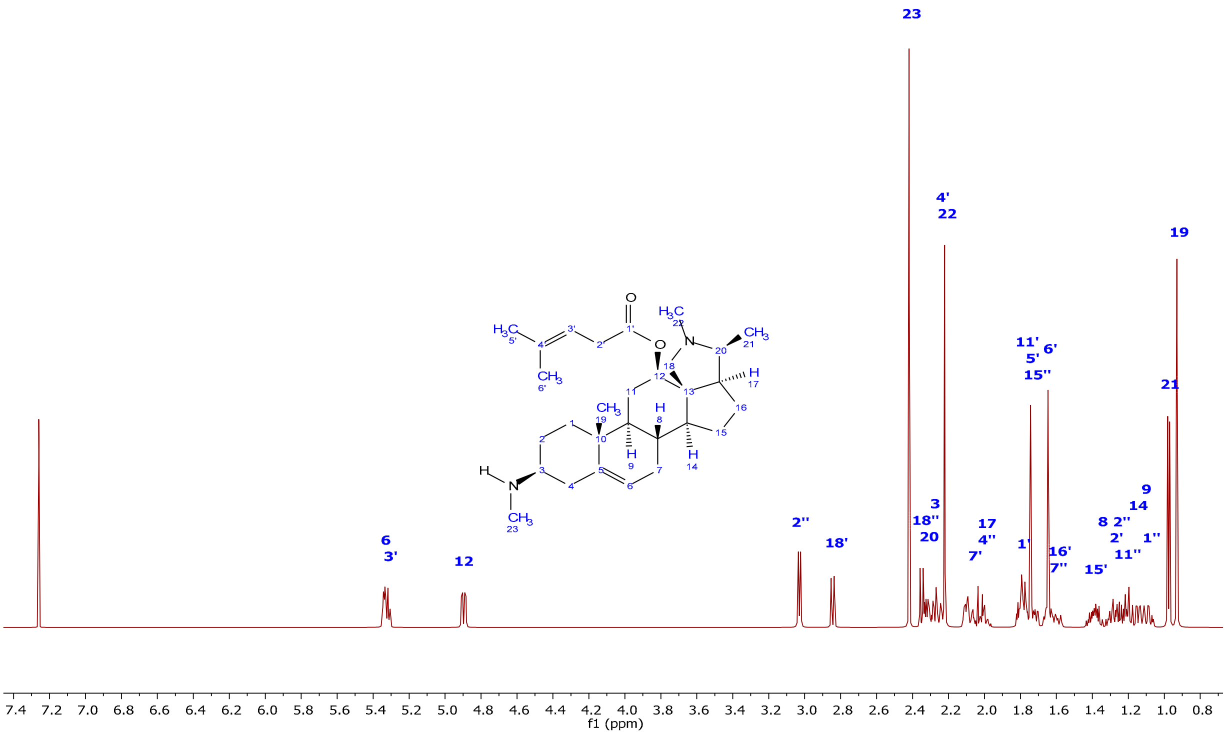

Figure S28: ^{1}H NMR spectrum of compound **14** ($CDCl_3$, 600 MHz)

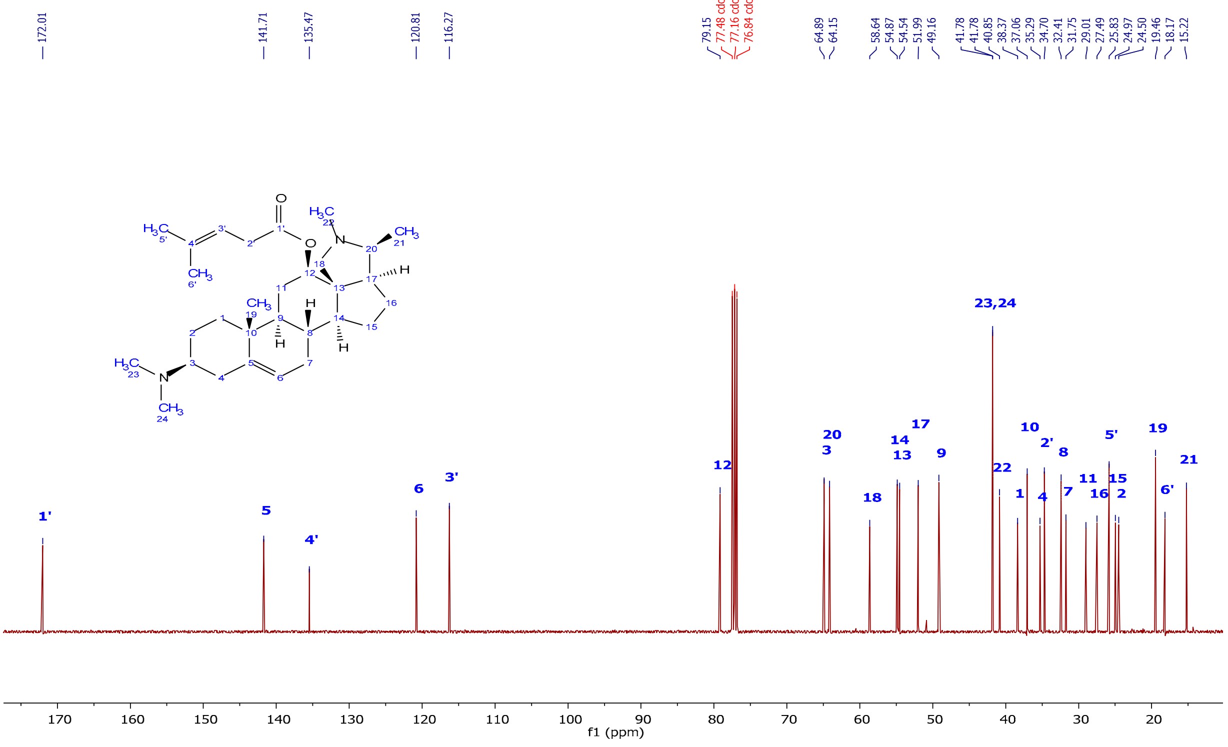

Figure S29: ^{13}C NMR spectrum of compound **15** ($CDCl_3$, 600 MHz)

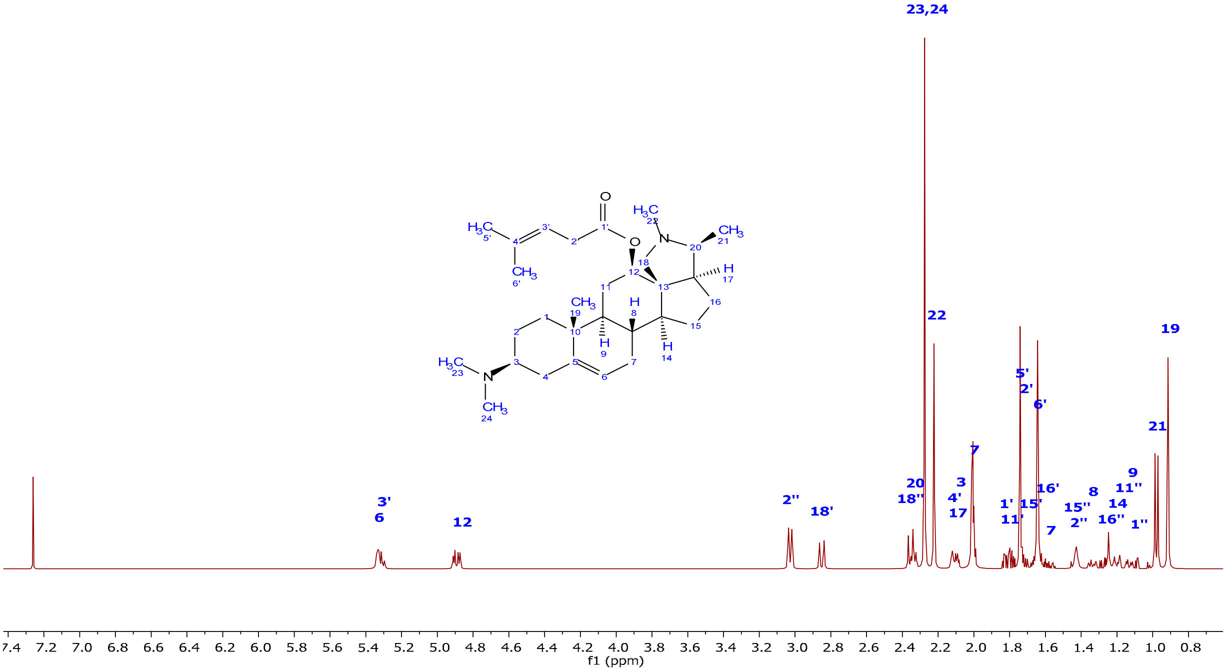

Figure S30: ^{1}H NMR spectrum of compound **15** ($CDCl_3$, 600 MHz)

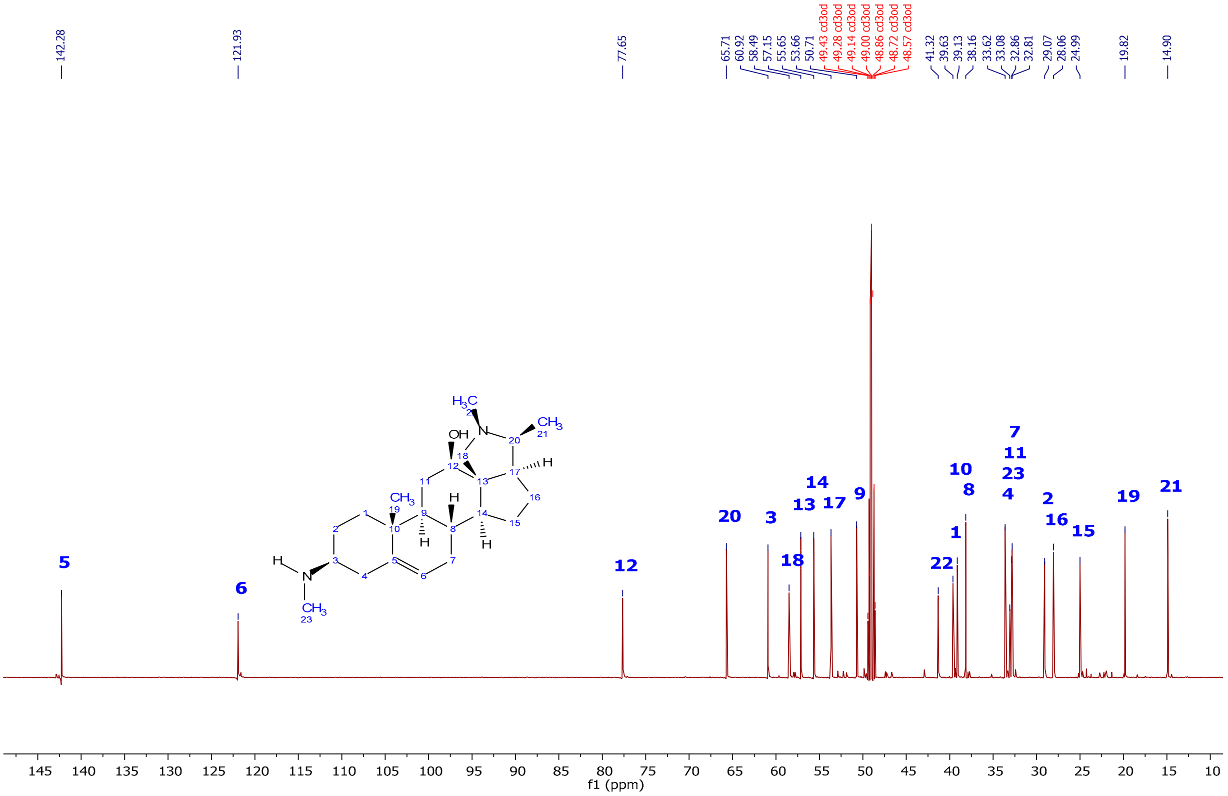

Figure S31: ^{13}C NMR spectrum of compound **16** (CD_3OD, 600 MHz)

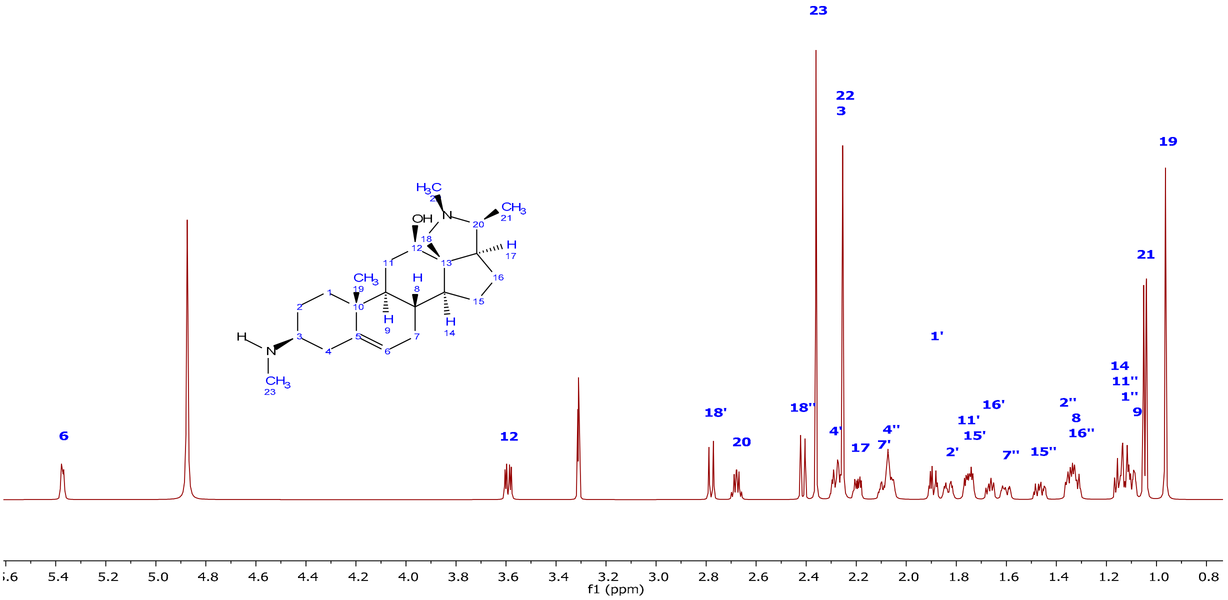

Figure S32: ^{1}H NMR spectrum of compound **16** (CD_3OD, 600 MHz)

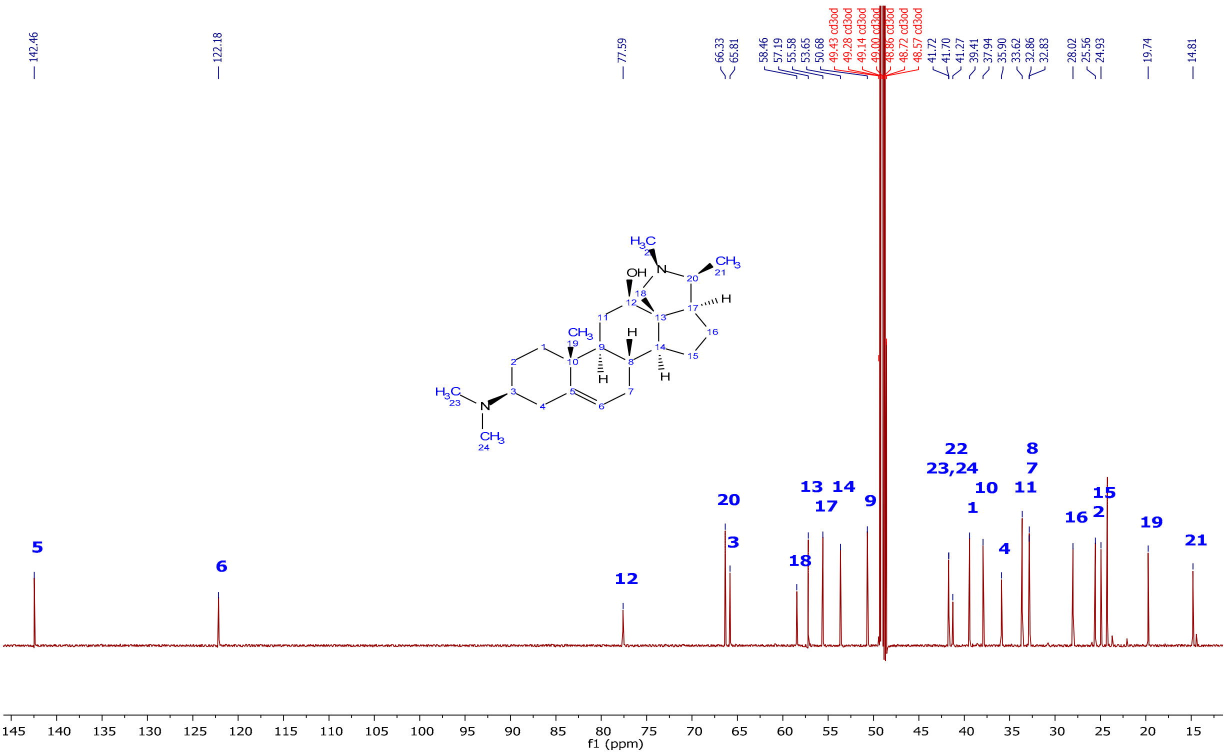

Figure S33: ^{13}C NMR spectrum of compound **17** (CD3OD, 600 MHz)

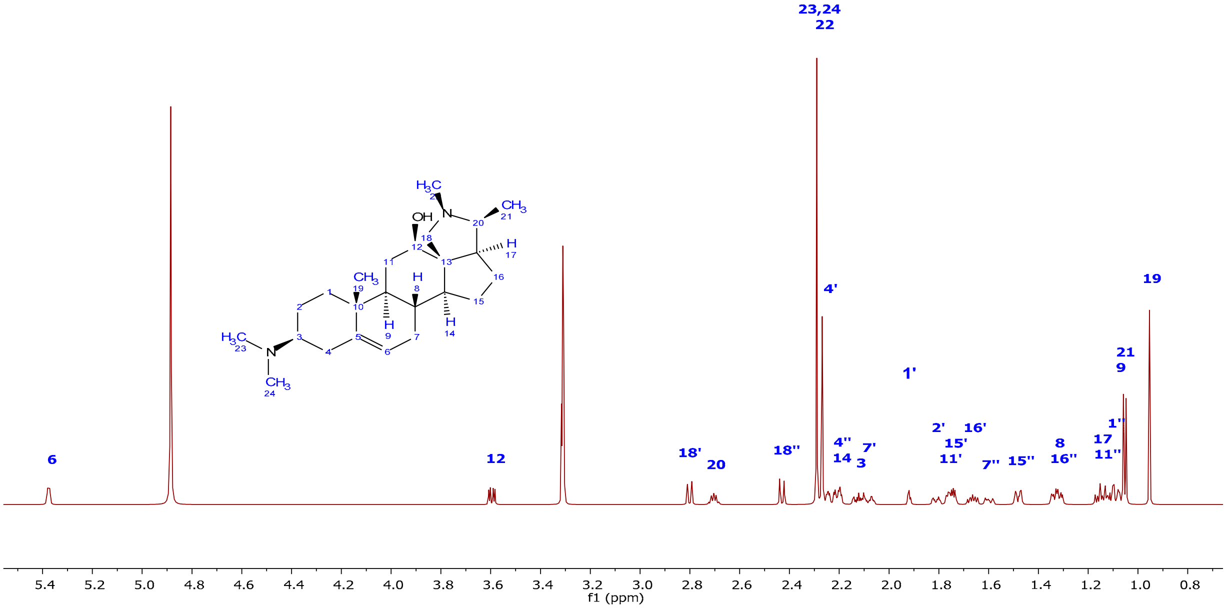

Figure S34: ^{1}H NMR spectrum of compound **17** (CD3OD, 600 MHz)

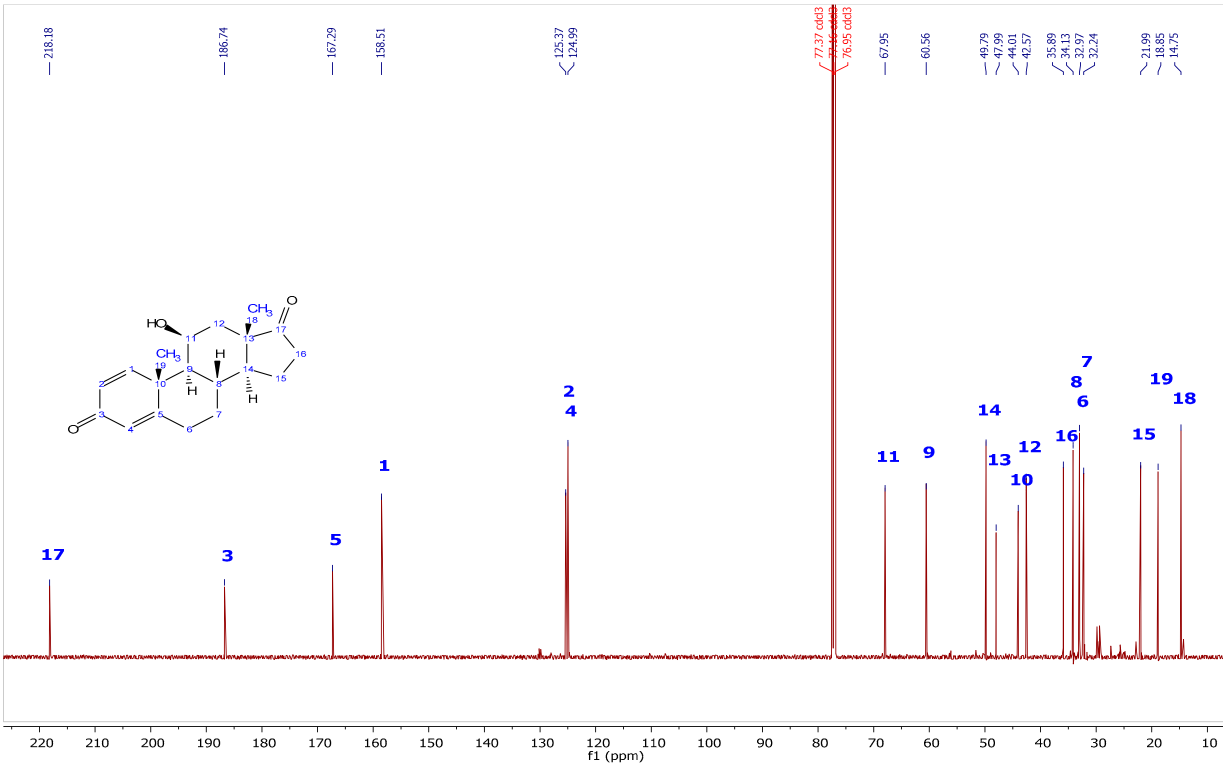

Figure S35: ^{13}C NMR spectrum of compound **18** ($CDCl_3$, 600 MHz)

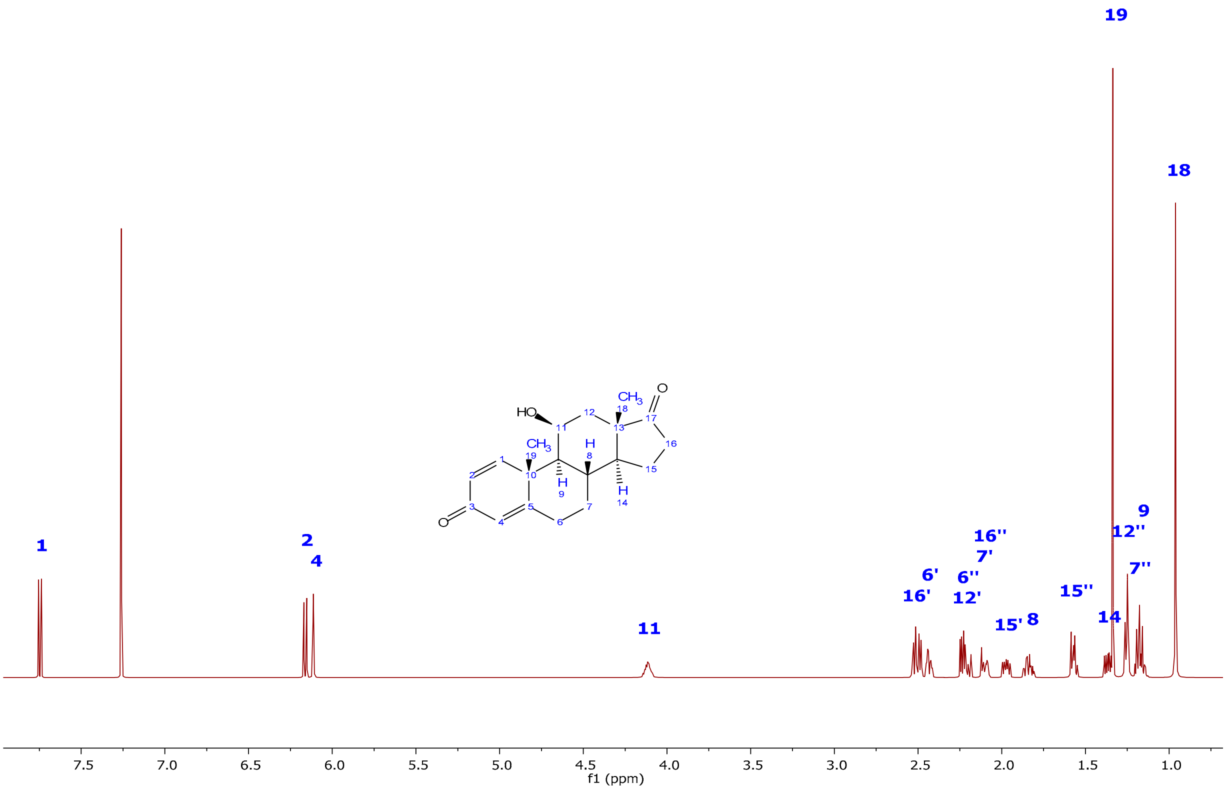

Figure S36: ^{1}H NMR spectrum of compound **18** ($CDCl_3$, 600 MHz)

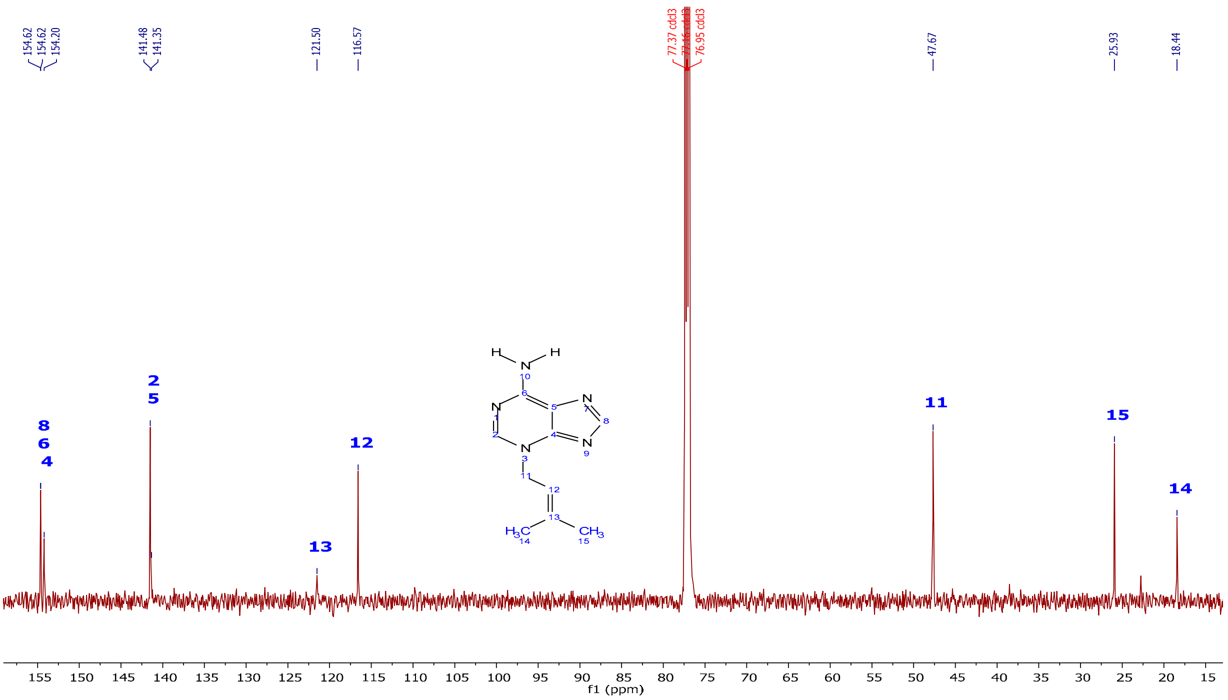

Figure S37: ^{13}C NMR spectrum of compound **19** ($CDCl_3$, 600 MHz)

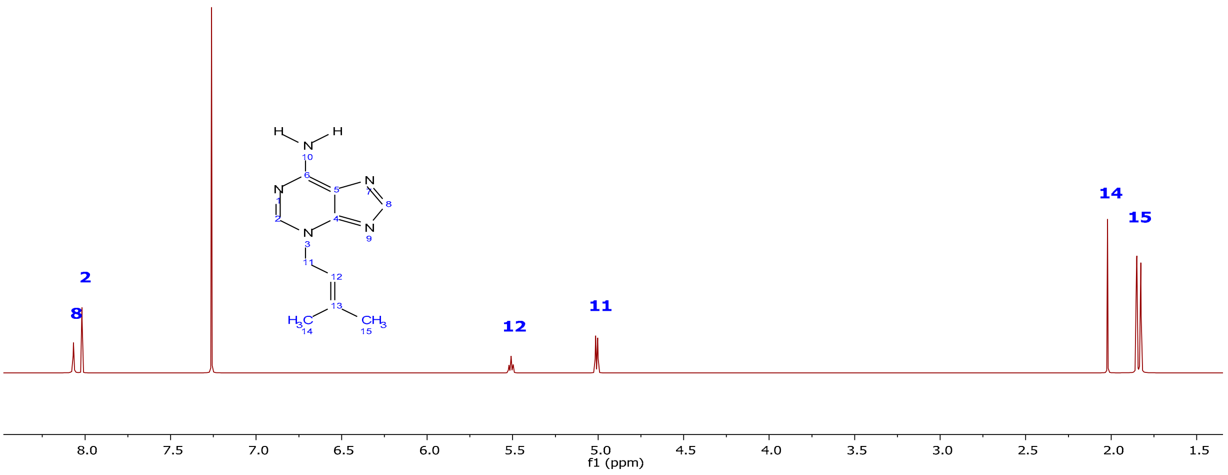

Figure S38: ^{1}H NMR spectrum of compound **19** ($CDCl_3$, 600 MHz)
